# DUSP5 regulated by YTHDF1-mediated m6A modification promotes epithelial-mesenchymal transition and EGFR-TKI resistance via the TGF-β/Smad signaling pathway in lung adenocarcinoma

**DOI:** 10.1186/s12935-024-03382-6

**Published:** 2024-06-13

**Authors:** Weina Fan, Ying Xing, Shi Yan, Wei Liu, Jinfeng Ning, Fanglin Tian, Xin Wang, Yuning Zhan, Lixin Luo, Mengru Cao, Jian Huang, Li Cai

**Affiliations:** 1 The Fourth Department of Medical Oncology, Harbin Medical University Cancer Hospital, Haping Road 150, Harbin, 150081 China; 2 Department of Thoracic Surgery, Harbin Medical University Cancer Hospital, Harbin, China

**Keywords:** EGFR-TKI resistance, Methylation, YTHDF1, DUSP5, Epithelial-mesenchymal transition, Metastasis

## Abstract

**Background:**

Lung adenocarcinoma (LUAD) patients have a dismal survival rate because of cancer metastasis and drug resistance. The study aims to identify the genes that concurrently modulate EMT, metastasis and EGFR-TKI resistance, and to investigate the underlying regulatory mechanisms.

**Methods:**

Cox regression and Kaplan–Meier analyses were applied to identify prognostic oncogenes in LUAD. Gene set enrichment analysis (GSEA) was used to indicate the biological functions of the gene. Wound-healing and Transwell assays were used to detect migratory and invasive ability. EGFR-TKI sensitivity was evaluated by assessing the proliferation, clonogenic survival and metastatic capability of cancer cells with treatment with gefitinib. Methylated RNA immunoprecipitation (MeRIP) and RNA immunoprecipitation (RIP) analyses established the level of m6A modification present on the target gene and the protein’s capability to interact with RNA, respectively. Single-sample gene set enrichment (ssGSEA) algorithm used to investigate levels of immune cell infiltration.

**Results:**

Our study identified dual-specificity phosphatase 5 (DUSP5) as a novel and powerful predictor of adverse outcomes for LUAD by using public datasets. Functional enrichment analysis found that DUSP5 was positively enriched in EMT and transforming growth factor-beta (TGF-β) signaling pathway, a prevailing pathway involved in the induction of EMT. As expected, DUSP5 knockdown suppressed EMT via inhibiting the canonical TGF-β/Smad signaling pathway in in vitro experiments. Consistently, knockdown of DUSP5 was first found to inhibit migratory ability and invasiveness of LUAD cells in in vitro and prevent lung metastasis in in vivo. DUSP5 knockdown re-sensitized gefitinib-resistant LUAD cells to gefitinib, accompanying reversion of EMT progress. In LUAD tissue samples, we found 14 cytosine-phosphate-guanine (CpG) sites of DUSP5 that were negatively associated with DUSP5 gene expression. Importantly, 5′Azacytidine (AZA), an FDA-approved DNA methyltransferase inhibitor, restored DUSP5 expression. Moreover, RIP experiments confirmed that YTH N6-methyladenosine RNA binding protein 1 (YTHDF1), a m6A reader protein, could bind DUSP5 mRNA. YTHDF1 promoted DUSP5 expression and the malignant phenotype of LUAD cells. In addition, the DUSP5-derived genomic model revealed the two clusters with distinguishable immune features and tumor mutational burden (TMB).

**Conclusions:**

Briefly, our study discovered DUSP5 which was regulated by epigenetic modification, might be a potential therapeutic target, especially in LUAD patients with acquired EGFR-TKI resistance.

**Graphical Abstract:**

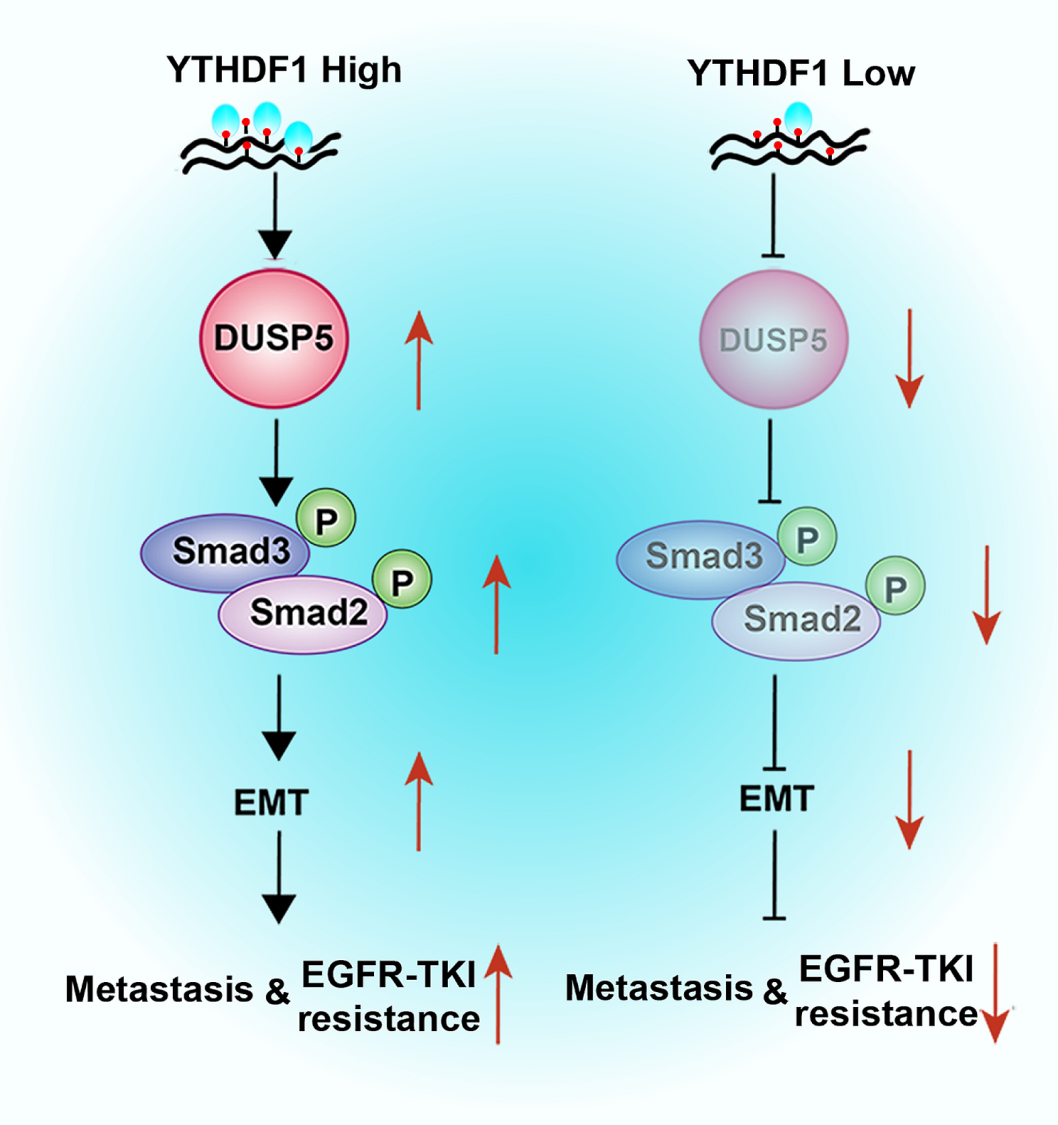

**Supplementary Information:**

The online version contains supplementary material available at 10.1186/s12935-024-03382-6.

## Introduction

Lung cancer is the deadliest type of tumor globally, remaining first in terms of mortality [1], and non-small cell lung cancer (NSCLC) is a primary type of lung cancer, constituting approximately 85% of all cases. As a predominant type of NSCLC, the Lung adenocarcinoma (LUAD) has a high propensity to metastasize to the distant organs and develop resistance to treatment, resulting in a grim five-year overall survival rate [2, 3]. Epidermal growth factor receptor tyrosine kinase inhibitors (EGFR-TKIs), such as gefitinib and erlotinib, are the conventional first-line therapy for advanced LUAD patients with sensitive epidermal growth factor receptor (EGFR) mutations and have demonstrated significant efficacy for these patients [4]. Unfortunately, the majority of LUAD patients experience incomplete responses, and eventually develop resistance to EGFR-TKIs within 9 to 13 months, which leads to the progression of the fatal disease [5]. The discovered acquired mechanisms of EGFR-TKI resistance have been largely classified as *EGFR* secondary mutations (e.g. EGFR-T790M mutation), bypass signaling activations, and phenotypic changes such as EMT [6]. This diversity and uncertainty in resistance mechanisms pose challenges to developing new therapeutic targets and predicting the effectiveness of EGFR-TKI treatment [7]. In conclusion, it is essential and attractive to discover and validate precise biomarkers for coupling modules of metastasis and EGFR-TKI resistance, offering a strategy to therapeutically combat the malignant progression of LUAD.

Epithelial-mesenchymal transition (EMT), a hallmark of tumorigenic transformation, is a cellular program where tumor cells lose their well-differentiated epithelial phenotypes and acquire invasive mesenchymal, fibroblast-like ones [8, 9]. Increasing evidence suggest that EMT is a critical contributor to cancer metastasis and drug resistance, including that to EGFR-TKIs [10]. During the sophisticated metastatic cascade of cancer, EMT progress is a prerequisite [11]. The drastically altered cancer cell surface during EMT prompts the re-localization or degradation of intercellular attachments, leading to the “leaky” tight junctions (TJs) and the destabilized adherence junctions (AJs) [12]. EMT lends metastatic cancer stem cells to survive, escaping from the primary site, and to invade and migrate to secondary areas [13]. Additionally, a growing number of studies disclosed that EMT or mesenchymal phenotype is correlated with both intrinsic and acquired resistance to EGFR-TKIs [14–16]. The molecular mechanisms by which the mesenchymal phenotype-mediated the EGFR-TKI resistance are still unknown [16]. Recent research suggested that mesenchymal cells might inherently exhibit reduced sensitivity to the inhibited intracellular signaling pathway, indicating a potential vulnerability to EGFR-TKIs [16–18]. For this dynamic and reversible process, EMT is strictly controlled by internal and external cues, including various regulators, effectors, and signaling pathways [19]. The transforming growth factor-beta (TGF-β) signaling pathway, in particular, has been presented to promote EMT, EMT-mediated metastasis and EGFR-TKI resistance [20]. Nevertheless, the mechanisms and factors that concurrently govern EMT-mediated metastasis and EGFR-TKI resistance remain largely unknown.

The rapid advancement of next-generation high throughput RNA sequencing technologies has resulted in the production of tremendous amounts of high-dimensional omics data [21]. Due to vast and reliable data sharing efforts by the research communities, such as the cancer genome atlas (TCGA), extensive and various omics data with unprecedented details are available [22, 23]. TCGA database and many datasets from the gene expression omnibus (GEO) provide detailed clinical information [24, 25]. In LUAD, according to the presence or absence of lymph node metastasis in the early pathologic T stage, Dong et al. identified macrophage-related secreted phosphoprotein 1 (SPP1) as a risk indicator for early lymph node metastasis across multiple cohorts, suggesting that SPP1 might function as a putative biomarker for the early detection of lymph node metastasis [26]. Gu et al. established a risk model comprising three differentially methylated genes, which possesses the ability to accurately predict prognosis in LUAD, based on the pathologic tumor node metastasis (TNM) stage [27]. However, the considerable volume of valuable omics data for LUAD metastasis and EMT had not been fully utilized and analyzed.

Herein, we first applied a novel combination of two public datasets (TCGA-LUAD and GEO: GSE11117) to identify distant metastasis-associated genes, and the seven differentially up-regulated genes were obtained. Among them, dual-specificity phosphatase 5 (DUSP5) exhibited an excellent ability to predict prognosis in multiple independent data sets. As indicated by functional enrichment analysis performed with GSEA, these experimental results validated that DUSP5 knockdown reversed EMT and inhibited the canonical TGF-β/Smad signaling pathway. Suppression of DUSP5 impeded tumor metastasis and EGFR-TKI resistance in LUAD cells. Meanwhile, YTHDF1 could bind DUSP5 mRNA to regulate its expression. Additionally, our clusters based on the DUSP5-originated genomic model exhibited distinct immune microenvironments, tumor mutational burden (TMB), and prognostic characteristics in LUAD. In short, the results revealed that DUSP5 acted as an influential mediator of EMT and could become a potential biomarker of prognosis and antitumor therapy of LUAD patients. Our results aimed to deliver a novel insight into the biological complexity related to metastasis and the resistance to targeted therapy, contributing to the tailored precision therapy for LUAD.

## Methods

### Data downloading and preprocessing

Transcriptome RNA-sequencing and clinical follow-up data were obtained from patients with TCGA-LUAD (https://portal.gdc.cancer.gov/projects). These following steps screened 482 TCGA-LUAD patients: (1) Retaining patients with clinical characteristics and OS time; (2) Keeping genes with Fragments Per Kilobase of exon model per Million mapped fragments (FPKM) greater than 1 in over 50% of the individuals. Furthermore, the data of GSE11117, GSE30219, GSE3141, GSE31210, GSE41271, GSE50081 and GSE72094 datasets were gained from GEO (https://www.ncbi.nlm.nih.gov/geo/).

### The analysis of differentially expressed genes (DEGs)

The “edgeR” R package was used to obtain DEGs from TCGA, with the threshold criteria consisting of a |Log_2_ fold change (FC)| greater than 0.585, false-discovery rate (FDR), and *P* < 0.05. The DEGs from GSE11117 were filtered using GEO2R (http://www.ncbi.nlm.nih.gov/geo/geo2r/) with a cutoff criteria of *P* < 0.05 and |Log_2_FC| greater than 0.585. DEGs were plotted as volcano plots based on bioinformatics (http://www.bioinformatics.com.cn/). PCA was used to visualize the discrimination between 2 groups (with distant metastasis and without distant metastasis) in terms of the R package “edgeR”, “ggplot2”, “ggrepel” and “FactoMineR”. A Venn diagram (https://hiplot-academic.com/basic/venn) was carried out to create interesting overlaps between two sets for identifying key targets.

### Analysis of cox regression and nomogram model

Exploring independent prognostic genes based on Cox regression analyses followed by using R, and these results showed as forest plots with the “survminer” R package. A nomogram prediction model was designed from these significant characteristics and genes obtained from multivariate Cox regression analysis. Then, we depicted the calibration plots to access the nomogram’s prognostic value.

### Survival analysis

Patients with TCGA-LUAD were categorized based on various clinical characteristics, which encompassed TNM stage (I/II and III/IV), T stage (T1/T2 and T3/T4), N stage (N0 and N1/N2/N3), M stage (M0 and M1), age (< 55 and ≥ 55 years old), and gender (male and female). In terms of optimal cutoff expression values, R tool was used to investigate OS in different cohorts by the Kaplan–Meier analysis. GEPIA analysis (http://gepia.cancer-pku.cn/) was used to assess the presence of DUSP5 in invasive breast carcinoma, cholangiocarcinoma, thyroid carcinoma, and esophageal carcinoma. The DUSP5 expression in various clinical features of TCGA-LUAD patients was calculated by Prism 8.0 (GraphPad, USA).

### LUAD cell culture, siRNA transfection and lentivirus infection

A549, H1299, HCC827, PC9, HCC827GR, and PC9GR cells were grown in RPMI1640 or DMEM medium supplemented with fetal bovine serum (FBS). The manufacturer’s instructions were followed for transient transfection with siRNA (Ribobio, Guangzhou, China) using Lipofectamine 3000 (Invitrogen). The following sequence: si1: GAGACTTTCTACTCGGAAT; si2: GTGGTAAATGTCAGCTACA. Lentiviral YTHDF1 shRNA-expressing (shYT-1 and shYT-2) vectors and control shRNA-expressing (NC) vectors were infected and selected by adding 2 µg/ml puromycin into medium.

### RNA immunoprecipitation (RIP) and methylated RNA immunoprecipitation (MeRIP)

A RIP Kit (Bes5101, BersinBio, China) was utilized to conduct the RIP assay. Input and coimmunoprecipitated RNAs were detected using qPCR.

Initially, the entire RNA was isolated using TRIzol reagent from TAKARA, a Japanese company. For the MeRIP assay, we utilized the riboMeRIPTM m6A Transcriptome Profiling Kit (C11051-1, Ribobio, China). Then the m6A-modified RNAs were analyzed for qPCR. SRAMP (http://www.cuilab.cn/sramp) was used to predict the MeRIP-qPCR primers for the target genes.

### Biological enrichment analysis

Genes with a correlation coefficient ≥ 0.3 were chosen to annotate using gene ontology (GO) enrichment analysis and Kyoto Encyclopedia of Genes and Genomes (KEGG) analyses in R software. Meanwhile, we visualized biological enrichment in bioinformatics (http://www.bioinformatics.com.cn/). The gene set named “h.all.v7.5.1.symbols.gmt” and gene set enrichment analysis (GSEA) (version 4.1.0) were utilized to identify hallmark pathways. Pathways with normalized *P* < 0.05 and FDR q value < 0.25 were considered significant. Additionally, the top 10 pathways were displayed based on the ranking of normalized enrichment scores (NESs).

### Obtainment of EMT-related genes

The Molecular Signatures Database v7.5.1 (MSigDB) (http://www.gsea-msigdb.org/gsea/msigdb) and dbEMT2 (http://www.dbemt.bioinfo-minzhao.org/browser.cgi#tsgene) provided access to EMT-associated genes. MSigDB contained 200 genes associated with EMT in the module labeled “HALLMARK_EPITHELIAL_MESENCHYMAL_TRANSITION”, while dbEMT2 contained 191 EMT-related genes.

### Single-cell analysis and Gene set cancer analysis (GSCALite)

In the CancerSEA (http://biocc.hrbmu.edu.cn/CancerSEA/), we also evaluated the functional conditions of DUSP5 in LUAD, including angiogenesis, apoptosis, cell cycle, differentiation, DNA damage, DNA repair, EMT, hypoxia, inflammation, invasion, metastasis, proliferation, quiescence, and stemness [28].

GSCALite is a foundational tool for cancer genomics analysis [29]. We examined the activation or inhibition of various pathways, including apoptosis, cell cycle, DNA damage response, EMT, hormone AR, hormone ER, PI3K/AKT, RAS/MAPK, RTK, and TSC/mTOR pathways, among the tools explored.

### Assessment of DNA methylation

TCGA has identified links between DNA methylation and gene expression through the use of MEXPRESS (https://mexpress.be/) [30] and SMART (http://www.bioinfo-zs.com/smartapp/) [31]. The MethPrimer software [32] was used to predict cytosine-phosphate-guanine (CpG) islands (CGIs) in the sequences of gene promoters. Meanwhile, for analyses of tumor vs. normal DNA methylation, we used the TCGA database. 5′Azacytidine (AZA) as a DNA methyltransferase inhibitor was purchased from MedChemExpress (HY-10,586).

### Western blotting

The proteins of cells were separated and transferred to polyvinylidene fluoride membranes using SDS-PAGE. After that, the primary antibodies DUSP5 (Abcam, ab200708), E-cadherin (Proteintech, 60335-1-lg), N-cadherin (Proteintech, 22018-1-AP), Vimentin (Abcam, ab92547), Snail (Cell Signaling Technology, #3879), Smad2 (Cell Signaling Technology, #5339), Smad3 (Cell Signaling Technology, #9523), P-smad2 (Cell Signaling Technology, #3108), P-smad3 (Cell Signaling Technology, #9520), YTHDF1 (Proteintech, 26787-1-AP) and GAPDH (Proteintech, 60004-1-lg) followed by, a secondary antibody was applied. The bands were imaged by a Tanon 5200 Imaging System (Tanon, China).

### Wound-healing and transwell assays

The cells were grown in 6-well dishes containing 4 × 10^5^ cells/ per well and then subjected to a scratch to create a wound resembling a crossroad. The serum-free 1640 was used to culture the cells after the scratch, and the migration of cells was imaged after 24, 36, or 48 h. ImageJ software was used to estimate wound closure rate.

Using Transwell assays to measure migratory and invasive capabilities via adding or not adding Matrigel coating. 2 × 10^4^ cells/well were placed in each well of the upper chamber using RPMI-1640. Additionally, 700 µL of RPMI1640 with FBS was introduced into the lower chambers. The enclosure was placed in an environment for a period of 24, 36, or 48 h. Following that, the cells were immobilized and colored using paraformaldehyde and crystal violet consecutively. Inverted microscopy was used to capture images.

### Cell counting kit (CCK-8), colony formation assays and EdU incorporation assay

In each well of 96-well plate, 3000 cells were cultured for 1–4 days. After incubating the CCK-8 reagent for 2 h, the absorption intensity at 450 nm was measured.

In the presence or absence of gefitinib therapy, a total of 1000 cells were grown in each well and then cultured in complete media for 12 days. Subsequently, the cells were fixed and stained sequentially with paraformaldehyde and crystal violet. Finally, they were adequately photographed and counted.

A total of 5000 cells were grown in a 96-well dish, followed by fixed and permeabilized using paraformaldehyde and Triton X-100. Subsequently, the cells were treated with EdU during incubation. Lastly, they were photographed by an inverted fluorescent microscope.

### Animal models

All animal experiments were approved by the Ethics Committee of Harbin Medical University. To establish the metastasis model in nude mice, six nude mice (Beijing Vitalstar Biotechnology, China) were randomly divided into two groups: NC and shDUSP5. Six nude mice were injected via the tail vein with 5 × 10^6^ A549-derived LUAD cells (NC and shDUSP5 cells). After 48 days, D-luciferin potassium salt was injected intraperitoneally at a dosage of 100 mg/kg to measure the bioluminescence signal from the tumors in the nude mice. The lungs of mice with metastatic tumors were promptly excised, treated with D-luciferin potassium salt, and the bioluminescence signal was measured again.

To establish EGFR-TKI sensitivity models in nude mice, ten mice were randomly divided into two groups: NC + Gefitinib and shDUSP5 + Gefitinib. We injected 1 × 10^7^ tumor cells subcutaneously into 4-week-old BALB/c mice. Once detectable tumors formed, the mice were administered gefitinib daily at a dosage of 100 mg/kg. After 28 days, the mice were euthanized. Tumor dimensions (long and short diameters) were measured using vernier calipers, and tumor weights were also recorded.

### Immune cell infiltration estimation

The ssGSEA analysis was conducted to quantify 29 different immune cell types in tissues based on normalized gene expression profiles by R. Wilcoxon test was used to examine the variations in infiltration levels. The TMB was assessed by the “maftools” package. We obtained 32 joint immune checkpoints in the literature [33], 20 (FPKM > 1 in more than 50% of the patients) of which were analyzed differentially.

### Statistical analysis

We analyzed our data with R language (version 4.1.3) and Prism 8.0 (GraphPad, USA). Differences were tested using the Student’s t-test and one-way ANOVA. *P* < 0.05 indicated statistically significant. The correlation was identified by Pearson’s correlation analysis in the bioinformatics website (http://www.bioinformatics.com.cn/) and Hiplot (https://hiplot-academic.com/basic/).

## Results

### DUSP5 is a novel independent prognostic predictor for LUAD

**Fig. 1:**
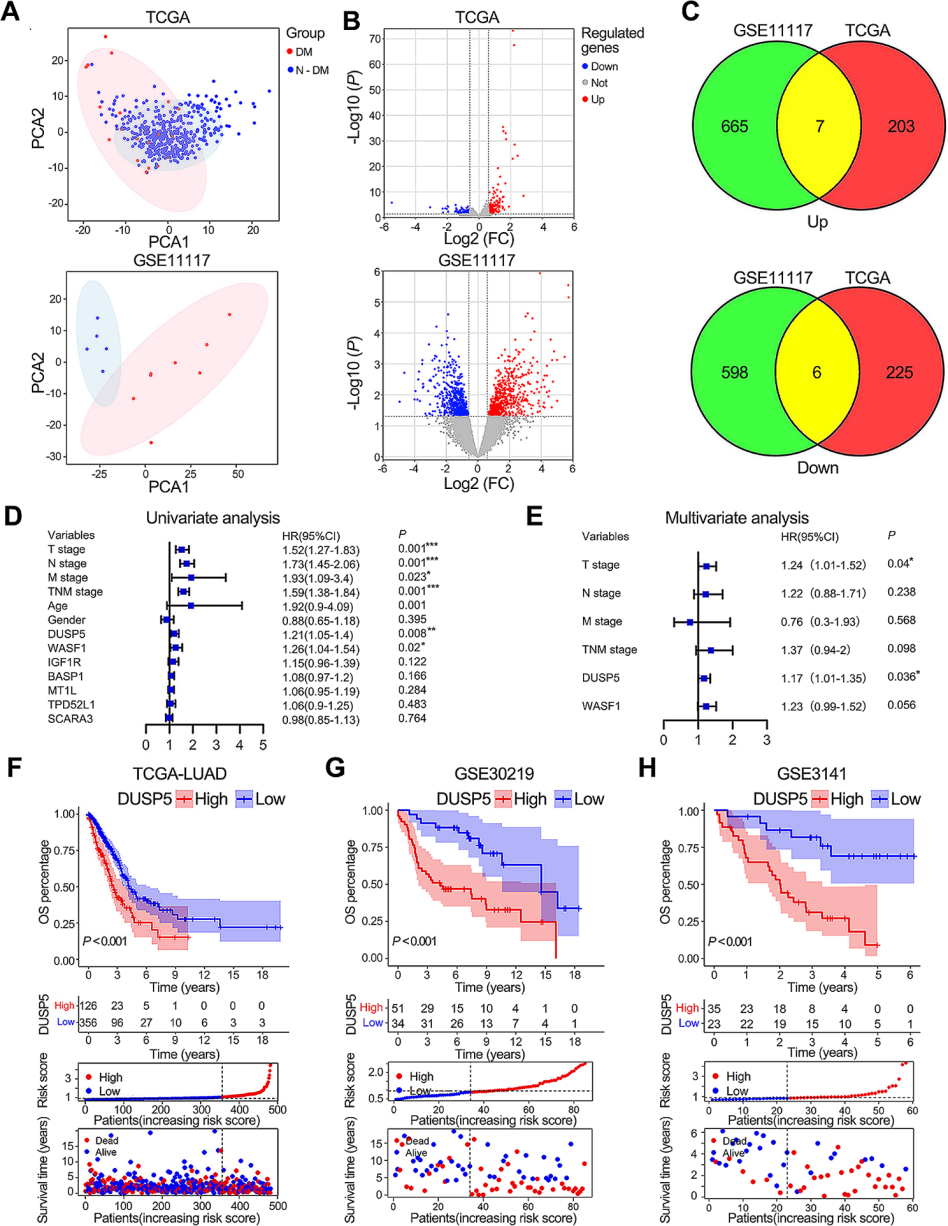
Identification of DUSP5 as an independent and poor prognostic indicator. (**A**) PCA for transcriptome profiles of 2 groups (DM: distant metastases and N-DM: no distant metastases), performing a difference on transcriptome between DM group and N-DM group. (**B**) Volcano plots exhibited these DEGs between the DM group and the N-DM group in TCGA and GSE11117. (**C**) Venn plots indicated 7 upregulated and 6 downregulated genes as intersected genes. (**D**, **E**) Univariate and Multivariate Cox regression analysis of genes and clinical features using the forest map. (**F**-**H**) Kaplan–Meier overall survival plot of DUSP5 based on TCGA, GSE30219 and GSE3141. FC: fold change; T stage: primary tumor stage; N stage: regional lymph node stage; M stage: distant metastasis stage

To identify distant metastasis-associated genes, we employed a new combination of two public datasets (TCGA-LUAD and GEO: GSE11117). Initially, we stratified patients from the TCGA-LUAD and GSE11117 datasets into cohorts with (DM) and without (N-DM) distant metastasis, based on their M stage. Using principal components analysis (PCA), we found apparent differences between DM and N-DM (Fig. 1A). Compared with N-DM, 210 genes were up-regulated in DM compared to N-DM, whereas 231 genes were down-regulated, based on the TCGA-LUAD database. 672 up-regulated genes and 604 down-regulated genes were discovered based on GSE11117 (Fig. 1B). As shown in Fig. 1C, Venn diagrams indicated seven up-regulated and six downregulated genes as the intersected genes. Next, univariate and multivariate cox-regression analyses were utilized to investigate the index-pendent tumor-promoting factor in the TCGA-LUAD dataset. In LUAD, DUSP5 and T stage were found to be independent risk biomarkers for poor prognosis (Fig. 1D, E).

To reliability, with put-back 100 times in advance, the median was used to randomly split 482 patients from the LUAD-TCGA dataset into two appropriate validation sets, preventing the random allocation bias. The clinical characteristics of these two groups were homogenously distributed (Table S1). The high DUSP5 expression could strongly predict dismal outcomes of LUAD patients in the two random TCGA validation sets, GSE30219 and GSE3141 datasets according to Kaplan–Meier overall survival (OS) curves (Fig. 1F-H, Figure S1A, B). In addition, using a clinical parameters-stratified analysis, such as T, N, M, TNM stage, gender and age, high DUSP5 expression also predicted poor prognosis in LUAD patient cohorts (Figure S2, S3). It is the first time that DUSP5 may contribute to the progression of LUAD.

### Expression levels of DUSP5 exhibit a positive correlation with T, M, and TNM stages

**Fig. 2:**
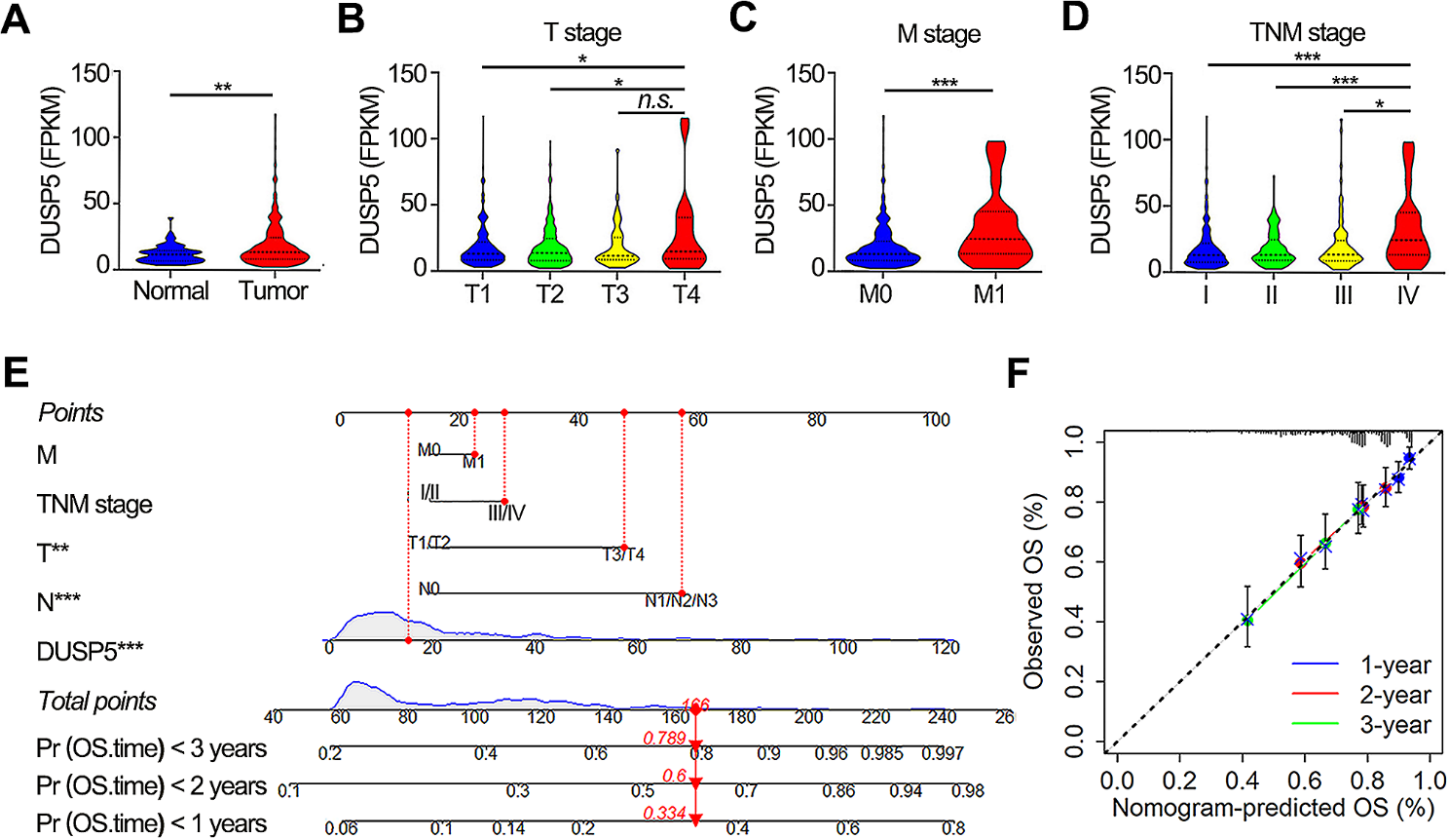
Increased DUSP5 expression is positively associated with clinicopathologic characteristics. (**A**) The levels of DUSP5 expression were examined between tumor and normal tissues according to data taken from the TCGA database. (**B**-**D**) Expression of DUSP5 in T stage, M stage and TNM stage based on TCGA database. (**E**) The nomogram predicted the probability of OS. (**F**) The calibration plot accessed the actual survival and predicted survival probability of OS. *P < 0.05, **P < 0.01, ***P < 0.001

DUSP5 expression was significantly elevated in tumor tissue samples than in normal lung tissue samples based on the TCGA-LUAD dataset (Fig. 2A). Moreover, we unearthed that the DUSP5 was upregulated in various cancers, including invasive breast carcinoma, cholangiocarcinoma, thyroid carcinoma, and esophageal carcinoma (Figure S4A). High DUSP5 expression was relative to the advanced pathologic T stage, M stage, and TNM stage (Fig. 2B-D). There was no relevance between the expression of DUSP5 and N stage, age, and gender (Fig. S4B-D).

For a good predictive model for clinical application, a nomogram was established according to total risk scores of DUSP5, T, N, M, and TNM stage (Fig. 2E). Through evaluation of the calibration curves which had the consistency of actual survival and predicted probability of 1-year, 2-year, and 3-year overall survival, we found that this model had excellent predictive accuracy (Fig. 2F).

### DUSP5 exhibited a positive correlation with metastasis, EMT, and TGF-β signaling pathway

KEGG pathway analysis exhibited that focal adhesion, TNF, MAPK, and NF-kappa B signaling pathways that were reported to govern cancer metastasis were listed as top 10 (Figure S5A) [34–37]. GO enrichment showed metastasis-associated biological processes, such as wound healing, epithelial cell migration, focal adhesion, and cell-cell junction enriched (Figure S5B) [38–42].

**Fig. 3:**
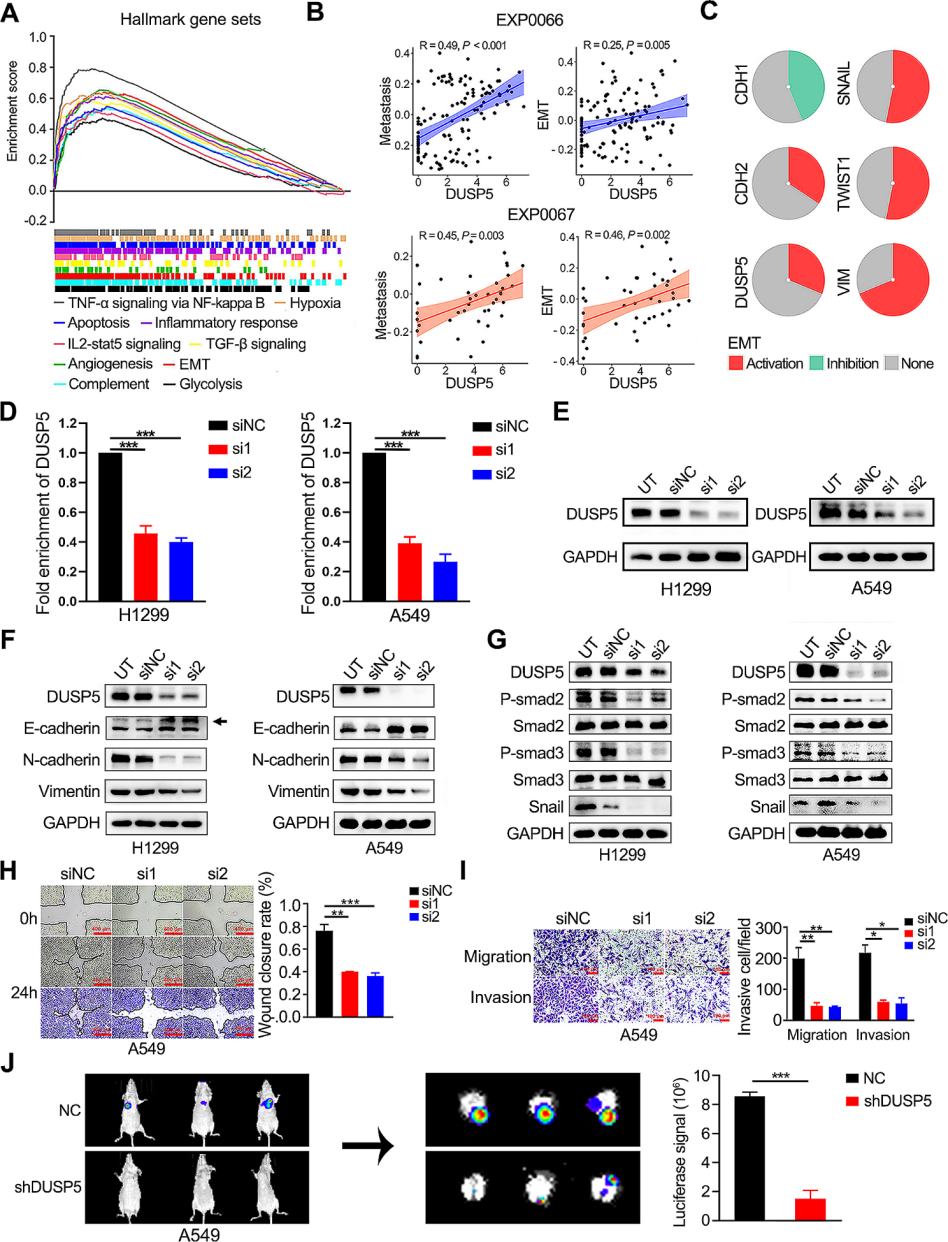
DUAP5 depletion inhibited EMT and TGF-β signaling pathways. (**A**) Hallmark analysis of DUSP5-related genes was performed by GSEA. (**B**) The correlation analysis between metastasis and DUSP5, and EMT and DUSP5 in the single-cell sequencing datasets (EXP0066 and EXP0067) of LUAD was based on the CancerSEA website. (**C**) The pie was used to represent the strength of genes (CDH1, CDH2, DUSP5, SNAIL, TWIST1 and VIM) of LUAD using GSCALite, red: activity; green: inhibition; grey: non-significant. (**D**) The RNA levels of DUSP5 expression in LUAD cells. (**E**)The protein levels of DUSP5 expression in LUAD cells were examined by Western blotting. (**F**) The levels of vimentin, N-cadherin and E-cadherin expression were assessed in UT, siNC, si1 and si2 cell lines. (**G**) The levels of P-smad2, Smad2, P-smad3, Smad3 and snail expression were assessed in UT, siNC, si1 and si2 cell lines. (**H**) Cell migratory capability was elucidated by a Wound-healing assay. (**I**) The invasive and migratory abilities were evaluated by Transwell assays. (**J**) The images display the bioluminescence signal intensities in each BALB/c nude mouse on day 48 after tail vein injection with NC or shDUSP5 cells (left panel). The bar charts present the results of quantitative and statistical analyses of the bioluminescence signal intensities for the NC and shDUSP5 groups (right panel). *P < 0.05, **P < 0.01, ***P < 0.001

Notably, GSEA was performed on *Hallmark* gene sets, EMT, and TGF-β signaling, the most prominent EMT inducer [43], as the top 10 *Hallmark* pathways were positively interrelated and remarkably enriched (Fig. 3A). In the two LUAD single-cell sequencing datasets, DUSP5 was also positively related to metastasis and EMT (Fig. 3B). Using GSCALite, DUSP5, CDH2, SNAIL, TWIST1, and VIM were associated with activated EMT progress, whereas CDH1 was involved in inhibited EMT progress (Fig. 3C, Figure S5C). As expected, 19 EMT-related genes from “MsigDB” and 8 oncogenic EMT-related genes from “dbEMT” correlated with DUSP5 expression with coefficients greater than 0.3 (Figure S6, S7A). In addition, EMT-related markers (VIM, FN1, SNAIL, SNAI2, TWIST1, and TWIST2) demonstrated elevated expression in the cohort characterized by high DUSP5 expression relative to the group with diminished DUSP5 expression levels (Figure S7B-C). Shortly, these data indicated that DUSP5 facilitated tumorigenesis and metastasis by inducing the EMT biological process in LUAD.

#### DUAP5 depletion inhibited metastasis, EMT and TGF-β signaling pathway

**Fig. 4:**
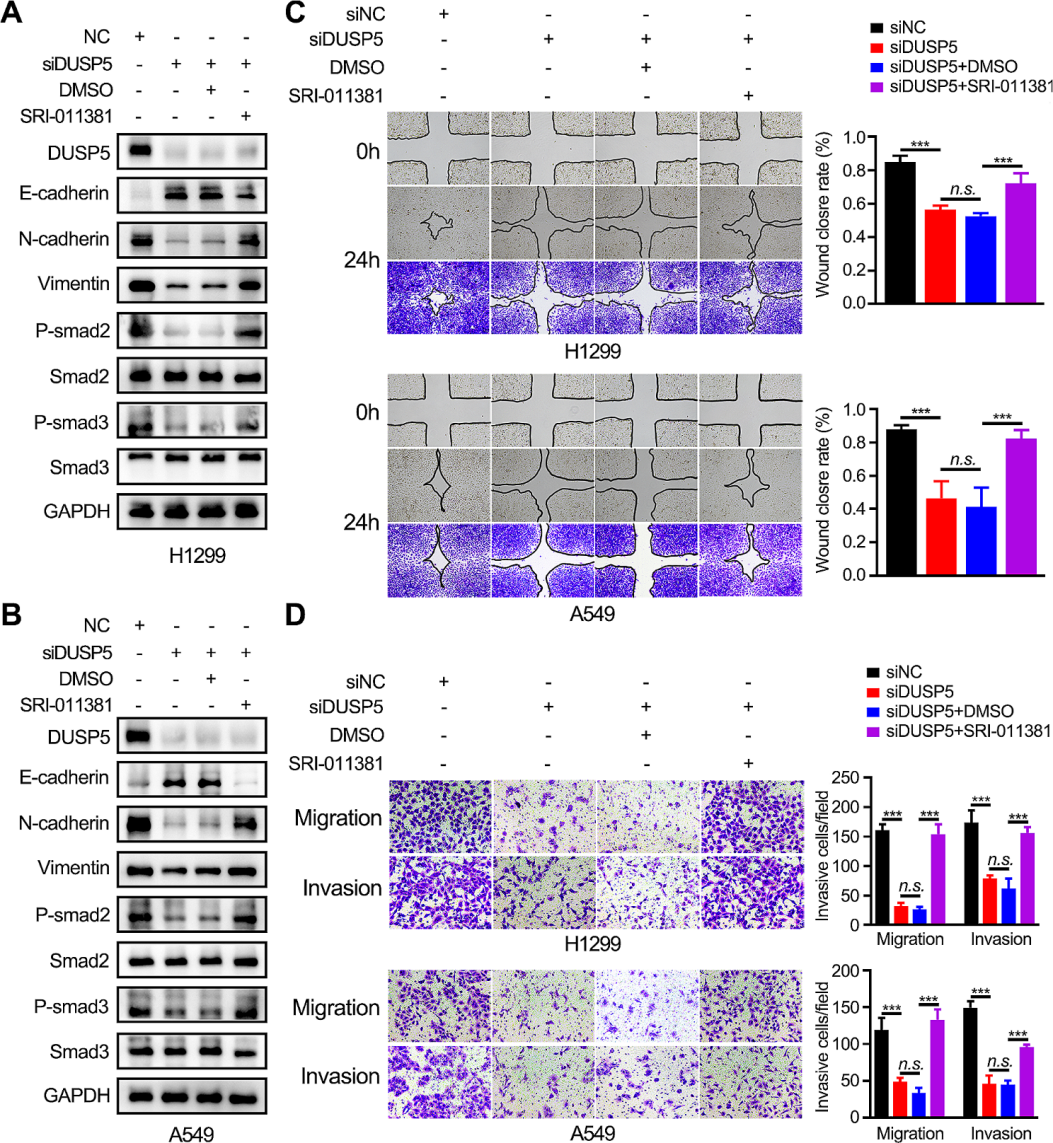
TGF-β signaling pathway is essential for DUSP5-mediated EMT and metastasis. (**A**, **B**) Western blot analysis validated the effect of treatment with SRI-011381 (10 µM) or DMSO for 24 h on the TGF-β signaling pathway and EMT after DUSP5 knockdown in LUAD cells. (**C**, **D**) The migratory and invasive ability of H1299 and A549 cells, with or without SRI-011381 treatment, was detected by Wound-healing assay and Transwell assays

To confirm the above bioinformatics results, we conducted loss-of-function experiments in which we exploited A549 and H1299 cell lines to silence DUSP5 expression using DUSP5-specific siRNAs (Fig. 3D-E). We found that the knockdown of DUSP5 enhanced E-cadherin expression and suppressed N-cadherin and vimentin expression in LUAD cells (Fig. 3F). In H1299 and A549 cells, the activation of the p-Smad2/3 and EMT-associated transcription factors snail were dramatically decreased by DUSP5 knockdown (Fig. 3G). According to Wound healing and Transwell assays, we verified that DUSP5 silence reduced the migratory and invasive ability of LUAD cells (Fig. 3H-I, Figure S8A-B). In the in vivo metastasis assays, knockdown of DUSP5 reduced the lung metastatic capability of LUAD cells in nude mice following tail vein injection, as indicated by lower luciferase signals. (Figure S8C, Fig. 3J). To verify whether DUSP5 regulated the EMT process and metastasis in LUAD through the TGF-β/Smad signaling pathway, we used SRI-011381, an administered agonist of the TGF-β signaling pathway. As shown in WB, SRI-011381 reversed the effects of the knockdown of DUSP5 resulting in the inhibition of P-smad2/3 and EMT (Fig. 4A, B). In addition, when treated with SRI-011381, DUSP5 knockdown failed to inhibit the migratory and invasive ability of LUAD cells (Fig. 4C, D). Together, our data indicated that DUSP5 is a driver for the EMT progression by activating the canonical TGF-β/Smad signaling pathway in LUAD.

### DUSP5 knockdown reversed EGFR-TKI resistance in gefitinib-resistant LUAD cells by inhibiting EMT

**Fig. 5:**
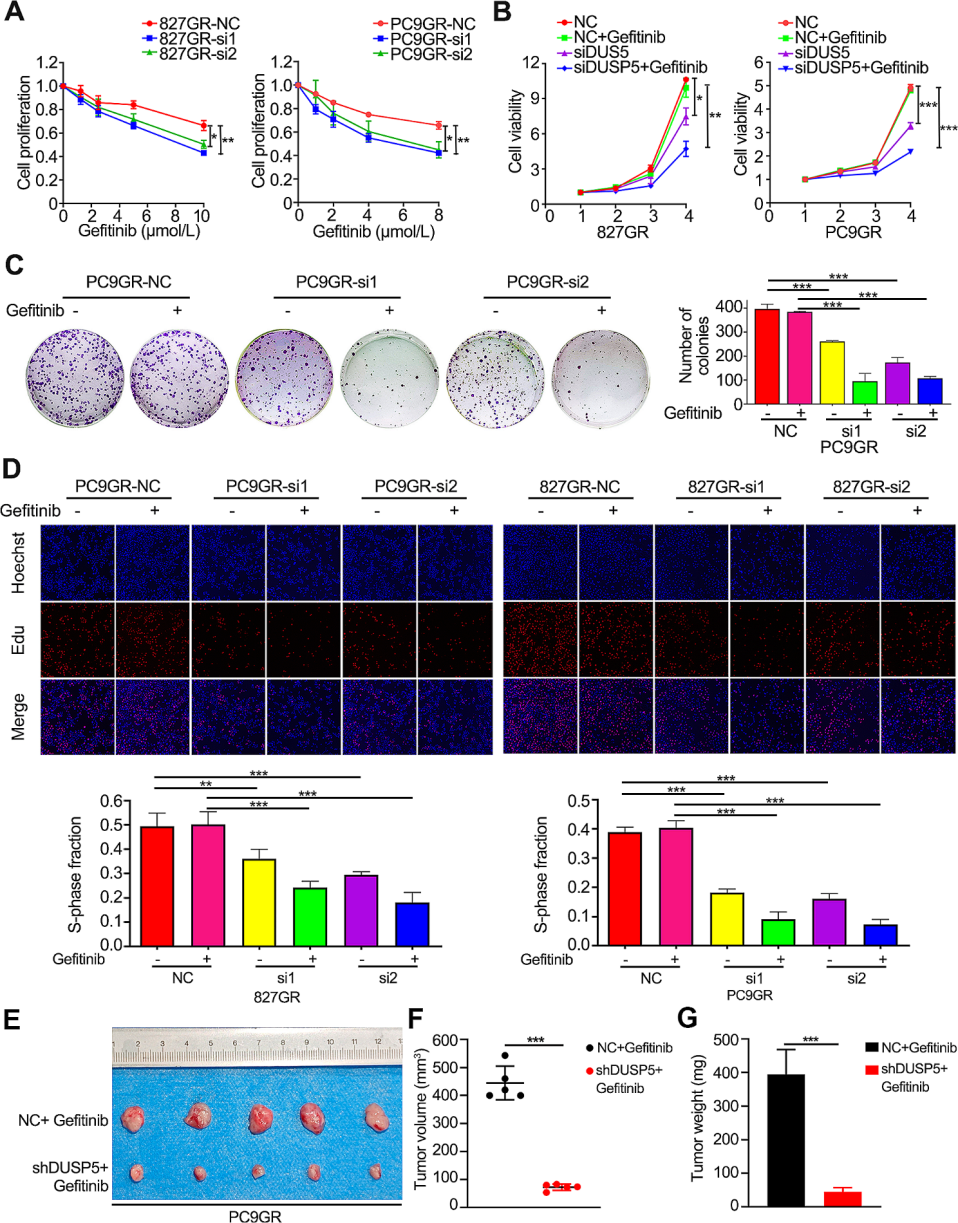
EGFR-TKI resistant LUAD cells could be re-sensitized by knocking down DUSP5 in vitro and in vivo. (**A**, **B**) CCK-8 analysis results performed the proliferation and viability of DUSP5 knockdown cell lines and EGFR-TKI resistant cell lines. (**C**, **D**) The proliferation of gefitinib-treated or untreated cells was detected by colony formation assay and EdU incorporation assay. (**E**-**G**) Panel E shows images of xenograft tumors from the two designated groups. Panels F and G depict the results of t-tests used to compare the mean tumor volumes (**F**) and weights (**G**) between the groups. *P < 0.05, **P < 0.01, ***P < 0.001

**Fig. 6:**
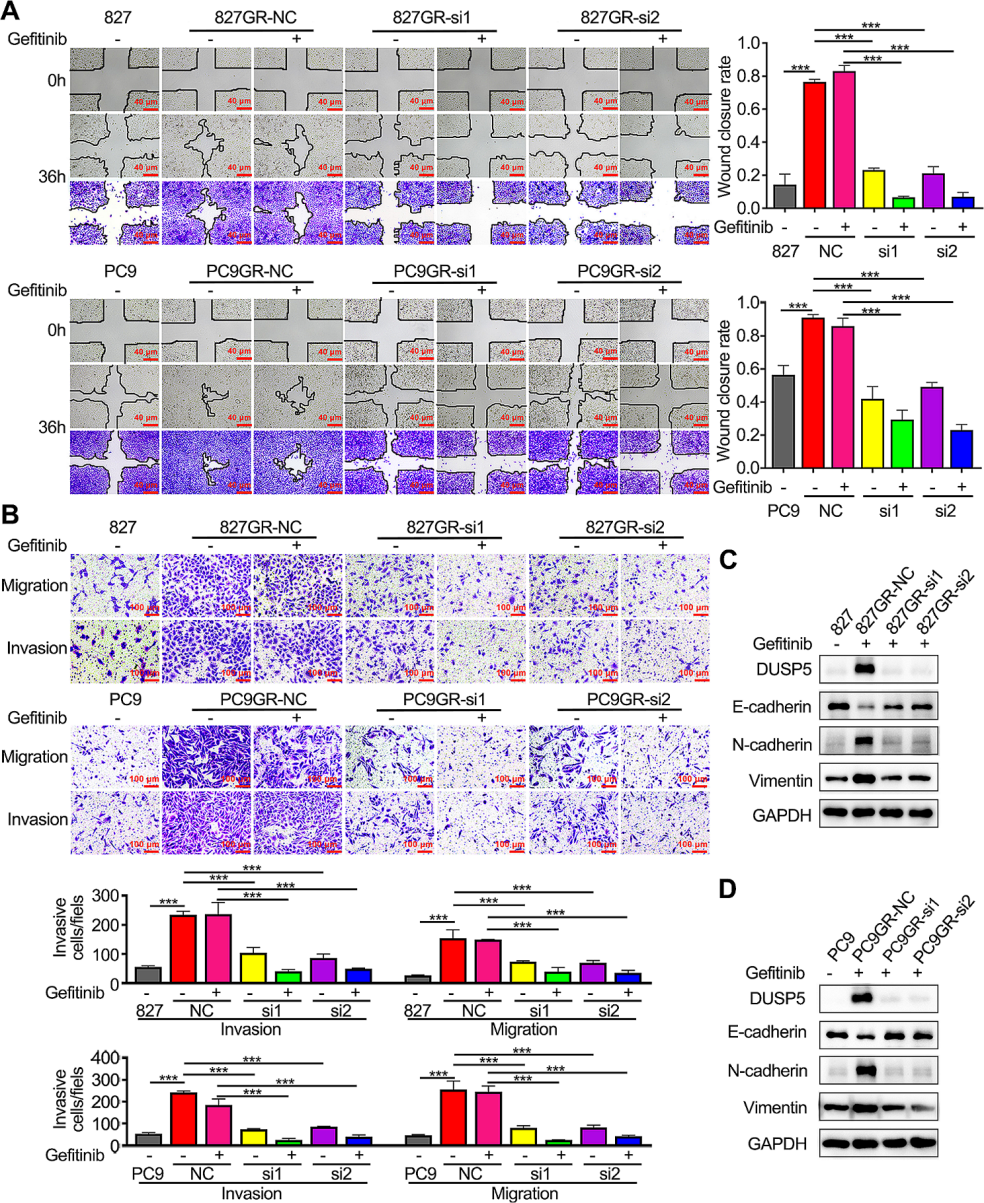
DUSP5 knockdown reverses EGFR-TKI resistance in gefitinib-resistant LUAD cells by inhibiting EMT. (**A**) The effect of DUSP5 knockdown on the motility of EGFR-TKI resistant cells was shown by Wound-healing assay. (**B**) The invasive and migratory abilities of EGFR-TKI resistant cells were assessed by Transwell assays after DUSP5 knockdown. (**C**, **D**) The levels of vimentin, N-cadherin and E-cadherin expression after DUSP5 knockdown. *P < 0.05, **P < 0.01, ***P < 0.001

EMT is recognized to facilitate EGFR-TKI resistance [44, 45]. To investigate if DUSP5 could induce drug resistance to EGFR-TKI, we transfected DUSP5-specific siRNAs into gefitinib-resistant cell lines (HCC827GR and PC9GR) to achieve DUSP5 knockdown (Figure S8E-F). Knockdown of DUSP5 re-sensitized gefitinib-resistant cell lines at different concentrations and treatment times of gefitinib (Fig. 5A-B). Interestingly, EGFR-TKI-resistant cell lines showed resistance to gefitinib, however, DUSP5 knockdown caused a significant decrease in cellular activity and inhibited the colony-forming ability of resistant cell lines (Fig. 5B-C, Figure S8G). Furthermore, similar results were obtained using the EdU incorporation experiments (Fig. 5D). In the in vivo gefitinib resistance experiment, xenografts (NC and shDUSP5) derived from PC9GR cells were established in nude athymic mice (Figure S8D). DUSP5 knockdown attenuated the gefitinib resistance in these cells, as evidenced by reduced tumor volume (Fig. 5E-F) and tumor weight (Fig. 5G). When treated with gefitinib, DUSP5 silence attenuated the metastatic abilities of EGFR-TKI-resistant LUAD cells; these effects were further enhanced when the combination of DUSP5 knockdown (Fig. 6A-B).

Of interest, examined by Western blotting, EGFR-TKI resistant cell lines displayed significantly lower abundance of E-cadherin, but higher expression of Vimentin and N-cadherin than parental cells (Fig. 6C-D). After DUSP5 knockdown, the mesenchymal phenotype in resistant cell lines was reversed, as indicated by increased E-cadherin and diminished N-cadherin and VIM expression (Fig. 6C-D). Collectively, our data indicated that DUSP5 decreased sensitivity to TKI treatment by inducing EMT.

#### DUSP5 was epigenetically regulated by DNA methylation and m6A modification

**Fig. 7:**
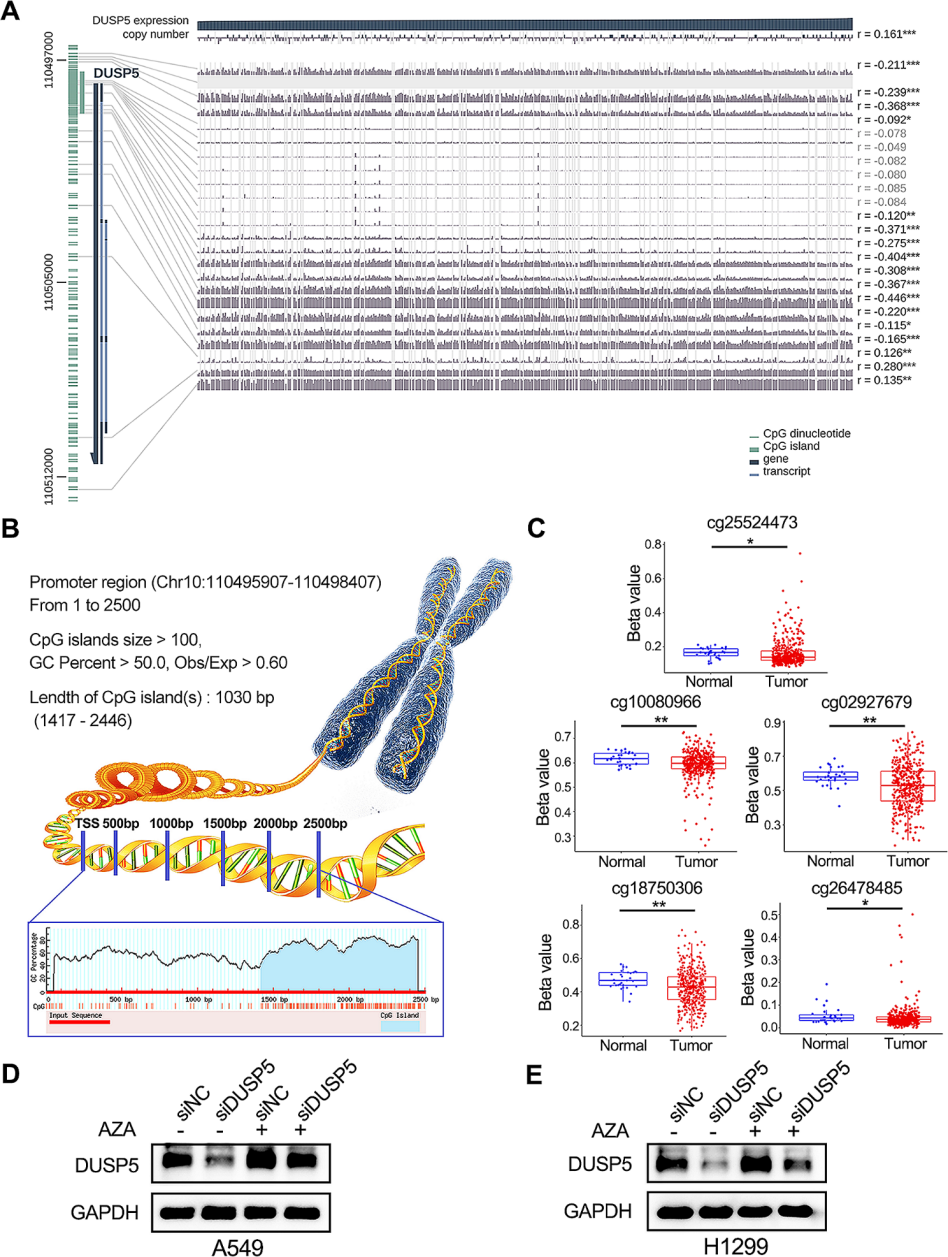
DUSP5 was epigenetically regulated by DNA methylation in LUAD. (**A**) The relationship between DNA methylation of the DUSP5 promoter region and DUSP5 expression with MEXPRESS. r: correlation coefficient. (**B**) Schematic representation of the CpG islands in the promoter region of DUSP5. (C) The beta value of CpG sites in tumor and normal tissue tissues. (**D**, **E**) The levels of DUSP5 expression were assessed with or without 1 µM AZA treatment. *P < 0.05, **P < 0.01, and ***P < 0.001

Methylation of DNA involves the dysregulation of gene expression [46]. Within the MEXPRESS database, fourteen CpG sites have been identified that exhibit an inverse correlation with the levels of DUSP5 expression (Fig. 7A). Using MethPrimer software, we obtained the CGI at the 1417–2446 sites in the promoter region of DUSP5, which meets the CGI criteria, including island size greater than 100 bp, GC percentage higher than 50.0, CpG dinucleotide Obs/Exp ratio greater than 0.6 [32] (Fig. 7B).

Next, we used the beta value to quantify methylation status. In comparison with normal tissue, the beta values in tumor tissue are high, indicating hypomethylation utilizing data from the TCGA-LUAD database (Fig. 7C). Further, the SMART website revealed that there were 11 significant correlations between the 22 CpG sites and the expression of DUSP5, including one positive correlation and ten negative correlations (|R| > 0.2, *P* < 0.05) (Figure S9). Consistently, AZA, a DNA methylation inhibitor, stimulated DUSP5 expression in LUAD cells, with or without depletion of DUSP5 (Fig. 7D, E). Our data suggest that DUSP5 was regulated by DNA methylation, and this regulation is negatively controlled by the degree of methylation of the DUSP5 promoter.

**Fig. 8:**
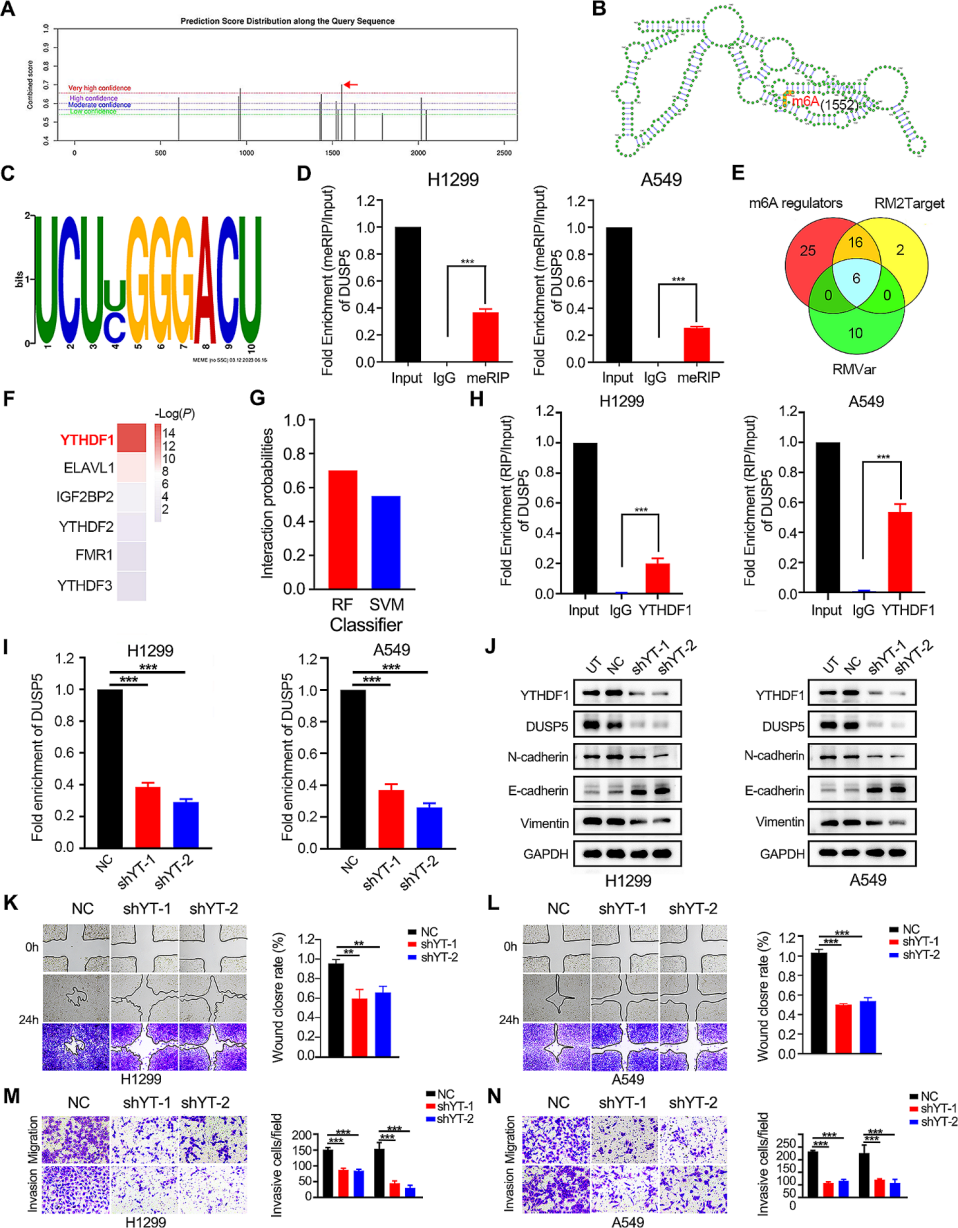
YTHDF1 is involved in the regulation of DUSP5 through the recognition of m6A methylation. (**A**) SRAMP website showed the potential sites of DUSP5 mRNA with m6A modification. The horizontal axis represented the position of the m6A modification sites, the vertical axis represented the combined score with m6A. (**B**) A graphical representation of the secondary structure context around the m6A modification sites of DUSP5 mRNA in SRAMP. The red dots represented sites of m6A modification. (**C**) m6A motif RRACH (D = A, G or U; R = A or G; H = A, U or C) was predicted by MEME. (**D**) MeRIP-qPCR showed that m6A levels of DUSP5 mRNA in H299 and A549 cells. (**E**) Venn plots displayed 6 intersected genes associated with m6A based on RM2target and RMVar. (**F**) Differential significance of six m6A-related genes between carcinoma and paracancer. (**G**) The interaction probability for DUSP5 and YTHDF1 predicted by RPISeq. (**H**) RIP-PCR showed that DUSP5 mRNA was immunoprecipitated by YTHDF1 protein. (**I**) The RNA levels of DUSP5 expression in LUAD cells. (**J**) Western blotting showed the DUSP5, Vimentin, N-cadherin and E-cadherin expression after YTHDF1 knockdown. (**K**-**L**) Cell migratory capability was elucidated by a Wound-healing assay. (**M**-**N**) The invasive and migratory abilities were evaluated by Transwell assays. **P < 0.01, and ***P < 0.001

In addition to the exploration of DNA modification of DUSP5, we further investigated its mRNA modification. The most common modification of mRNA is m6A [47]. Using the online bioinformatic tools, SRAMP and MEME, the typical m6A motif RRACH (D = A, G or U; R = A or G; H = A, U or C) was identified in DUSP5 mRNA based on GSE136433 (Fig. 8A-C, Figure S10A-B). MeRIP-qPCR experiments revealed that DUSP5 underwent significant m6A methylation in LUAD cells (Fig. 8D). Using RM2Target [48] and RMVar [49] online databases, we predicted six m6A regulators that might modify DUSP5 mRNA m6A (Fig. 8E). Among them, YTHDF1 was most significantly overexpressed in LUAD tissues at the optimal *P*-value (Fig. 8F, Figure S10C).

Furthermore, we anticipated the robust interactions between YTHDF1 and DUSP5 (probability score > 0.5 was considered positive) via RPISeq approaches (Fig. 8G). Then, RIP experiments revealed that DUSP5 mRNA was immunoprecipitated by the YTHDF1 protein (Fig. 8H). We successfully knocked down DUSP5 (Fig. 8I). Western blotting confirmed that YTHDF1 silence inhibited DUSP5 expression and EMT progress (Fig. 8J). Consistently, the knockdown of DUSP5 resulted in decreased migration and invasion abilities of LUAD cells (Fig. 8K-N). These data suggested that YTHDF1 could facilitate the DUSP5-mediated EMT process and metastasis through m6A modification.

#### Construction of the DUSP5-originated genomic model

We identified 111 DUSP5-originated genes in LUAD patients using the limits of |FC| >2 and FDR < 0.05 (Figure S11A). GO annotations data and KEGG analysis showed that DUSP5-originated genes were linked with many malignant biological behaviors and oncogenic signaling pathways, such as epithelial cell migration and MAPK signaling pathway (Figure S11B, C). Using univariate-Cox regression models, we discovered that 69 DUSP5-originated genes can predict the prognosis of LUAD patients (Table S2). Then 69 genes were taken into the least absolute shrinkage and selection operator (LASSO) regression analysis to establish a DUSP5-originated genomic model for LUAD patients. Three prognosis-related genes were identified, including, Gap junction protein, beta 3 (GJB3), family with sequence similarity 83 member A (FAM83A), and fascin actin-bundling protein 1 (FSCN1) (Figure S11D, E). The LUAD patients were stratified into two cohorts predicated upon the median value of the risk score (Figure S11F). The three genes were all strongly expressed in the high-risk group (Figure S11G). LUAD patients who belonged to the high-risk group had a remarkably worse OS (Figure S11H). As observed in Figure S11I, the DUSP5-originated genomic model had the highest C-index among these prognostic factors including risk score, and T, N, M, and TNM stage.

#### Verification of prognostic significance of DUSP5-originated genomic model

The predictive value of this DUSP5-originated genomic model was verified among external datasets. The high-risk group could forecast a worse outcome in the GSE30219, GSE31210, GSE41271, GSE50081, and GSE72094 datasets (Figure S12A-E). Furthermore, the risk score of the DUSP5-originated genomic model could predict poor prognosis following cox-regression analysis in the TCGA-LUAD dataset (Figure S12F, G). To produce an accurate predictive model for therapeutic application, a nomogram based on the risk score, T, N, M, and TNM stage was built (Figure S12H). The calibration curves revealed that this model had excellent prediction accuracy (Figure S12I).

### The DUSP5-originated genomic model revealed two clusters with distinct immune features

We then investigated the variations in levels of immune cells between various groups to comprehend the relationships between the DUSP5-originated genomic model and the immune microenvironment (TME). ssGSEA algorithm revealed that 16 of the 28 immune infiltrating cells varied between two groups, with the high-risk group showing higher infiltration of immune cells, indicating an immunological “hot” phenotype. On the contrary, the low-risk group displayed an immunological phenotype known as “cold” (Figure S13A). Additionally, we found the expression of immune checkpoint genes, including PDCD1 (PD-1), CD274 (PD-L1), IL23A, JAK1, LDHA, LAMA3, PDCD1LG2, PVR, TNFRSF18, TNFRSF4, and TNFRSF9, were higher in the high-risk group compared with that in the low-risk group (Figure S13B, Figure S14).

As shown in Figure S13C, the 15 genes, the most frequently mutated proto-oncogene or tumor suppressor gene, had a high rate of mutations (93.19% and 87.29%) in the two groups, respectively. TMB is an accurate predictive biomarker for immune checkpoint blockade (ICB) therapy, and patients with higher TMB may suggest better immunotherapy efficiency [50]. Our data revealed disclosed an augmented tumor mutational burden (TMB) within the high-risk cohort, suggesting an enhanced potential responsiveness to immunotherapeutic interventions (Figure S13D). Additionally, survival analysis elucidated that the patients within the high-TMB cohort exhibited a superior OS compared to their counterparts in the low-TMB cohort (Figure S13E). Further, the Kaplan-Meier survival curve demonstrated that the cohort characterized by a high TMB concomitant with a low-risk score manifested the most favorable five-year survival prognosis, whereas the group with a low TMB and high-risk score presented the least favorable prognosis (Figure S13F). Consequently, patients in the high-risk group exhibited an increased likelihood of deriving clinical benefit from ICB therapy.

## Discussion

**Fig. 9:**
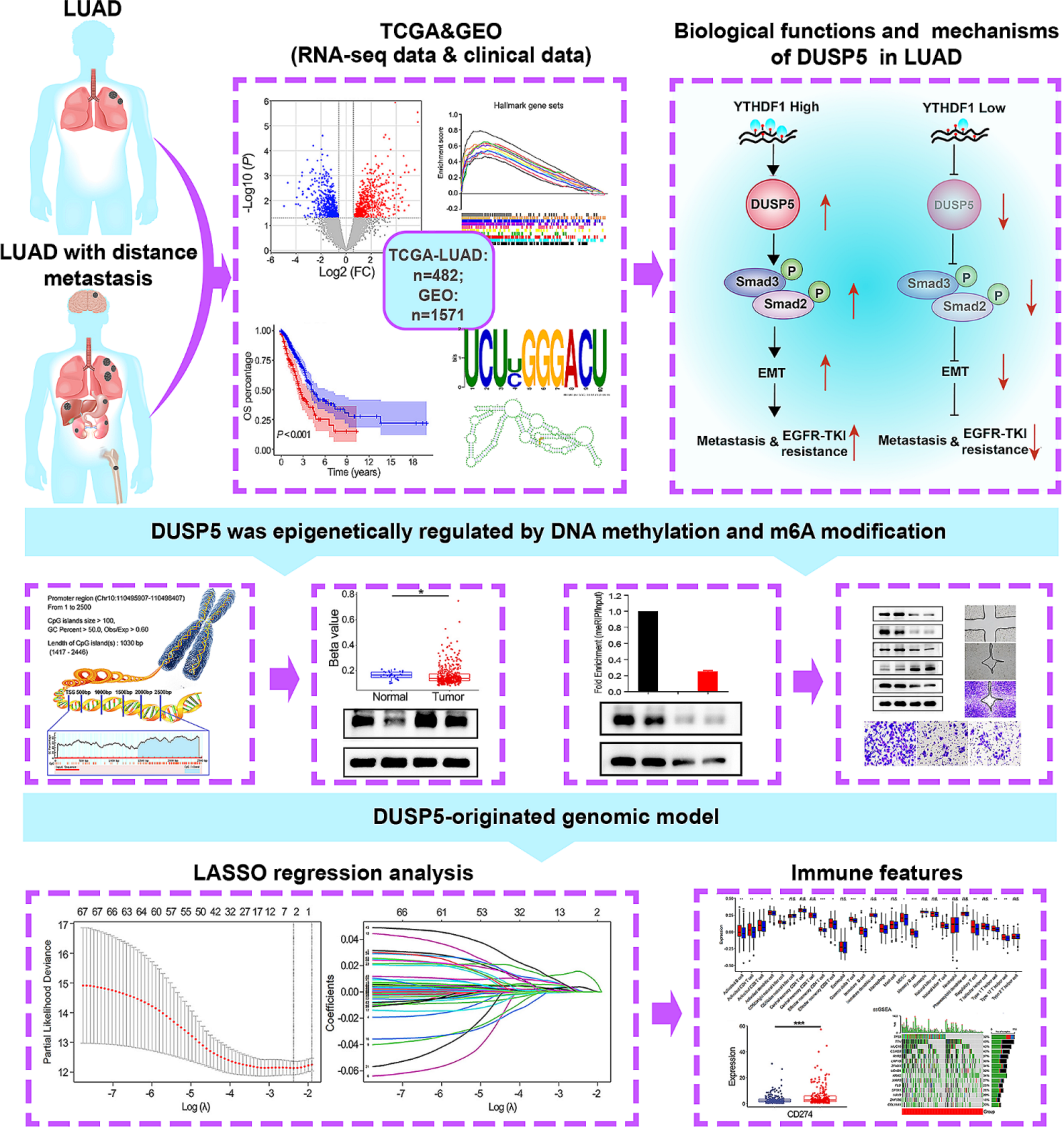
The flow chart of this study

The majority of LUAD patients succumb to tumor metastasis, therapeutic resistance including EGFR-TKI treatment, and relapse of disease, rather than their primary tumor [51, 52]. The relevant players and mechanisms have not been sufficiently investigated. We first combined the two public datasets (TCGA-LUAD and GEO: GSE11117) and performed an integrative analysis, identifying DUSP5 with robust prognostic significance and its functions in the EMT and TGF-β signaling pathway. Experimentally, we found that suppression of DUSP5 significantly inhibited the TGF-β signaling pathway-mediated EMT, thus impeding tumor metastasis and EGFR-TKI resistance. The DUSP5 expression was first postulated and then validated to be regulated by DNA methylation. Meanwhile, we have discovered that YTHDF1 could bind DUSP5 mRNA and regulate DUSP5 expression. Then, the two clusters with distinct immune features and TMB were revealed by the DUSP5-derived genomic model (Fig. 9).

For the first time, DUSP5 was demonstrated to result in LUAD progression in this study. The kinase-interacting motif of DUSP5 facilitates its capacity to catalyze the dephosphorylation of threonine/serine as well as tyrosine residues within its substrate molecules [53]. DUSP5 dephosphorylate toward extracellular signal-regulated kinase (ERK), contributing to nuclear translocation and inactivation of ERK [54]. It is reasonable that both DUSP5 and ERK are “double-edged sword” [55, 56]. DUSP5 is overexpressed in human papillary thyroid carcinomas (PTCs), and DUSP5 silencing suppresses the migratory ability and invasiveness of PTCs cells [57]. Consistently, *Olaia et al.* disclose that DUSP5 was a potential biomarker of poor prognosis, and they speculate that DUSP5 may prevent ERK1/2-mediated tumor development in human neuroblastoma [58]. In line with these findings, our data suggested a pro-tumorigenic role of DUSP5 by both experimental and bioinformatic approaches in LUAD. On the contrary, DUSP5 is found to be a tumor-suppressive factor by inactivation of ERK in gastric cancer, colorectal cancer and so on [59–63]. The reason that DUSP5 functions as an oncogene in LUAD is not investigated here but warrants further exploration.

Our findings indicate that DUSP5 augments resistance to EGFR-TKI and fosters the EMT process by mediating the activation of the TGF-β/Smad signaling pathway in LUAD. TGF-β signaling pathway is the most well-known pathway that controls the EMT process [64]. The canonical TGF-β signaling pathway involves TGF-receptor I kinase directly phosphorylating Smad2/3 before activated Smad2/3 binds with Smad4 to create a complex that translocates into the nucleus and augments the expression of downstream target genes [65]. Non-canonical TGF-β signaling pathways involve JNK, Ras-ERK, PI3K-Akt pathway, and so on [66]. Whether DUSP5 regulates non-canonical TGF-β signaling pathways to modulate EMT in LUAD remains unknown and further exploration is needed. So far, several inhibitors of the TGF-β signaling pathway have been used in preclinical and clinical stages, such as TβRII-Fc (ligand traps), AP12009 (antisense oligonucleotides), SB-431,542 (small molecule receptor kinase inhibitors), Trx-SARA (peptide aptamers) [67, 68]. Importantly, it has been hypothesized that neoplastic cells are induced to initiate EMT as a mechanism to acquire resistance to EGFR-TKIs [69]. Our research outcomes have the potential to inform the advancement of enhanced precision therapeutic strategies for patients with LUAD, particularly those who are most likely to benefit from interventions targeting the TGF-β signaling axis or EGFR-TKIs.

Herein, DNA methylation can control DUSP5 gene expression. DNA methylation, histone modification, nucleosome remodeling, and RNA interference represent exemplars of epigenetic regulatory mechanisms [70]. DNA methylation is the most well-characterized and reversible epigenetic modification to date [71, 72]. Generally, it encompasses the enzymatic conveyance of a methyl moiety to the fifth carbon atom of the cytosine nucleotide, resulting in the formation of 5-methylcytosine in genomic DNA by DNA methyltransferases (DNMTs) [73]. This change modifies chromatin structure, interactions with DNA proteins, DNA structure, stability, and gene expression [74]. In accordance with our study, we observed hypomethylation of DUSP5 in LUAD cells, which resulted in the upregulation of DUSP5 expression. AZA is a well-known, highly effective anticancer medication that is used to treat acute myeloid leukemia, melanoma, breast cancer, and colon cancer [75–80]. AZA promotes DNA demethylation by inhibiting DNMTs during replication, inducing silence of gene expression [81, 82]. According to our results, DUSP5 may function as a putative biomarker for directing the stratification of patients with a heightened propensity for responsiveness to AZA.

Here, our findings delineate, for the inaugural instance, that DUSP5 is subject to regulation via YTHDF1-mediated m6A modification. Through a variety of molecular mechanisms, including enhancing protein translation and modifying mRNA stability, YTHDF1 controls the expression of target genes [83]. According to the previous study, YTHDF1 is responsible for promoting translation initiation and subsequent protein translation [84]. Our results of RIP experiments suggested that YTHDF1 modulates DUSP5 expression by altering mRNA stability. Similar mechanisms have previously been reported [85–87]. Zhao et al. elucidated that YTHDF1 enhances the stability of c-Myc mRNA catalyzed by METTL3, thereby augmenting the expression of c-Myc [85]. . Additionally, YTHDF1 knockdown dramatically reduces the half-life of HK2 mRNA, suggesting that YTHDF1 may play a role in maintaining HK2 RNA stability [86]. YTHDF1, through its interaction with the m6A-modified 5’UTR of the mRNA, enhances the translation elongation and mRNA stability of PDK4 by recruiting eukaryotic translation elongation factor 2 (eEF-2) and insulin-like growth factor 2 mRNA-binding protein 3 (IGF2BP3) [87]. The specific mechanisms by which YTHDF1 governs DUSP5 expression require further exploration.

Tumor-infiltrating immune cell levels, the expressions of immune checkpoints including PD-1, PD-L1 and CTLA-4, and TMB were found to be elevated in the high-risk group relative to the low-risk group, as classified by the genomic model-derived from DUSP5. Immunotherapy, including ICB therapy, has brought revolutionary changes to the management of LUAD [88, 89]. Immune infiltrations are the primary targets of the immunotherapy [90]. Immune “hot” tumors with more infiltrating immune cells are more likely to be responsive to immunotherapy than immune “cold” tumors with a lower density of infiltrating immune cells [91]. Moreover, immune checkpoint molecules and TMB have been proven to be reliable biomarkers to predict the response of immune checkpoint blockade therapy [92, 93]. Thus, our DUSP5-originated genomic model for LUAD could help to appraise a predictor of response to ICB treatment strategies and display robust performance on survival prediction.

## Conclusions

In summary, we first demonstrated that DUSP5 is a novel prognostic biomarker and pro-oncogenic gene for LUAD. Our data indicated DNA hypomethylation and YTHDF1-mediated aberrant increased expression of DUSP5 orchestrates metastasis and development of EGFR-TKI resistance in LUAD by inducing TGF-β/Smad signaling pathway-mediated EMT. Our results suggested that the involvement of the DUSP5 and DUSP5-derived genes might be utilized to guide a personalized approach to metastatic patients and patients with target therapy and immunotherapy in LUAD.

### Electronic supplementary material

Below is the link to the electronic supplementary material.

Supplementary Material 1

Supplementary Material 2

Supplementary Material 3

Supplementary Material 4

Supplementary Material 5

Supplementary Material 6

Supplementary Material 7

Supplementary Material 8

Supplementary Material 9

Supplementary Material 10

Supplementary Material 11

Supplementary Material 12

Supplementary Material 13

Supplementary Material 14

Supplementary Material 15

Supplementary Material 16

Supplementary Material 17

## Data Availability

Data is available on reasonable request. All data generated or analyzed during this study are available from either the supplementary information or the corresponding author.

## Abbreviations

EMT: Epithelial-mesenchymal transition
EGFR: Epidermal growth factor receptor
EGFR-TKIs: Epidermal growth factor receptor tyrosine kinase inhibitors
LUAD: Lung adenocarcinoma
TCGA: The Cancer Genome Atlas
GEO: Gene Expression Omnibus
GSEA: Gene set enrichment analysis
MeRIP: methylated RNA immunoprecipitation
RIP: RNA immunoprecipitation
ssGSEA: Single-sample gene set enrichment
DUSP5: Dual-specificity phosphatase 5
YTHDF1: YTH N6-methyladenosine RNA binding protein 1
AZA: 5′Azacytidine
TMB: Tumor mutational burden
NSCLC: Non-small cell lung cancer
TJs: Tight junctions
AJs: Adherence junctions
SPP1: Secreted phosphoprotein 1
m6A: N6-methyladenosine
FPKM: Fragments Per Kilobase of exon model per Million mapped fragments
FC: Fold change
FDR: False-discovery rate
FBS: Fetal bovine serum
NESs: Normalized enrichment scores
MSigDB: Molecular Signatures Database v7.5.1
GSCALite: Gene Set Cancer Analysis
CGIs: CpG islands
DM: With distant metastasis
N-DM: Without distant metastasis
PCA: Principal components analysis
FAM83A: Family with sequence similarity 83 member A
GJB3: Gap junction protein, beta 3
FSCN1: Fascin actin-bundling protein 1
TME: The immune microenvironment
ICB: Immune checkpoint blockade
ERK: Extracellular signal-regulated kinase
PTCs: Papillary thyroid carcinomas
DNMTs: DNA methyltransferases
