# Supplementary figures and images for: DUSP5 regulated by YTHDF1-mediated m6A modification promotes epithelial-mesenchymal transition and EGFR-TKI resistance via the TGF-β/Smad signaling pathway in lung adenocarcinoma
Source: Cancer Cell Int. 2024 Jun 13;24:208. doi: 10.1186/s12935-024-03382-6 (PMC11177384; doi:10.1186/s12935-024-03382-6)

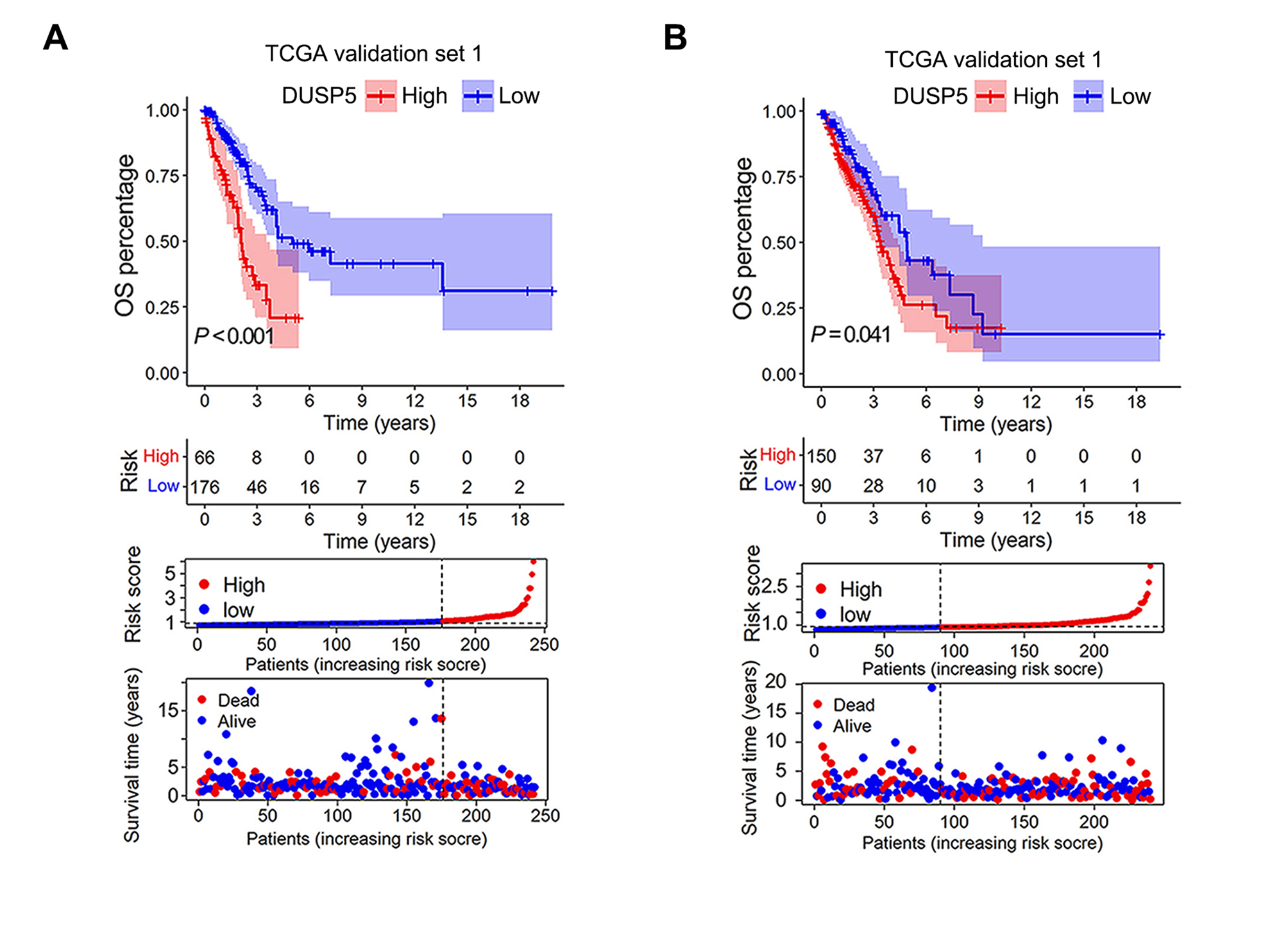

Supplement: Supplementary file 1 — Supplementary Material 1 [file 12935_2024_3382_MOESM1_ESM.tif]

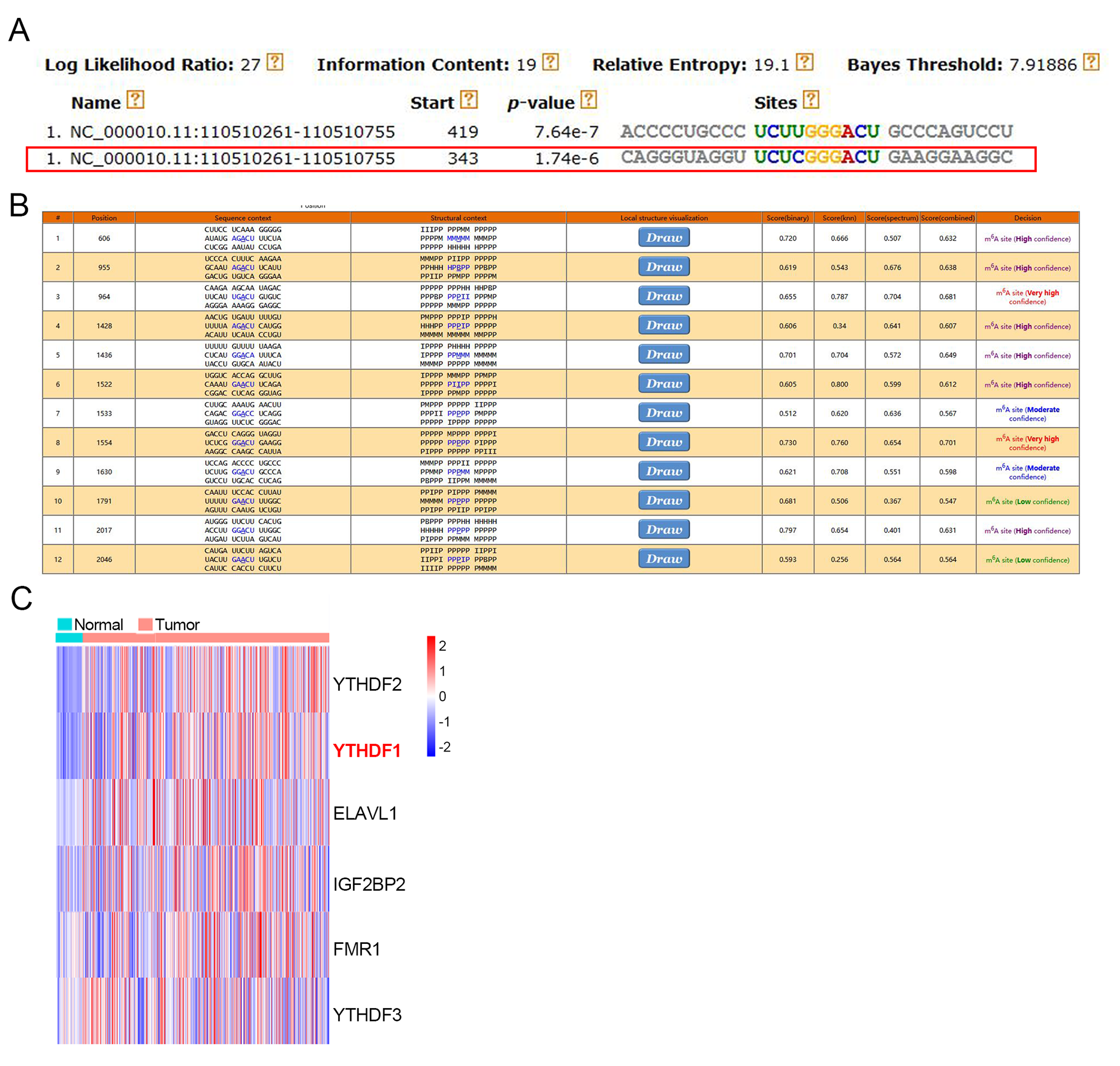

Supplement: Supplementary file 2 — Supplementary Material 2 [file 12935_2024_3382_MOESM2_ESM.tif]

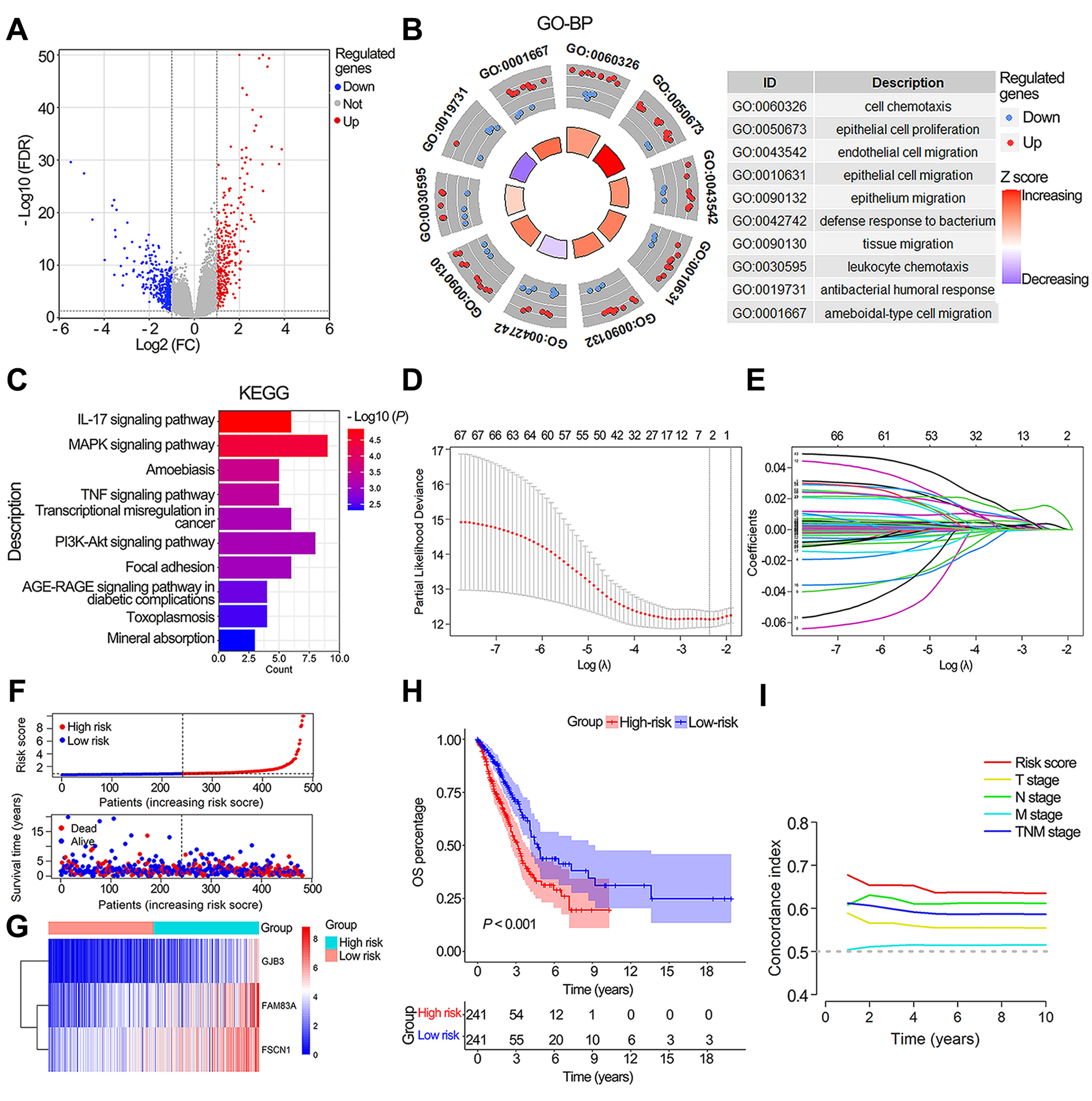

Supplement: Supplementary file 3 — Supplementary Material 3 [file 12935_2024_3382_MOESM3_ESM.tif]

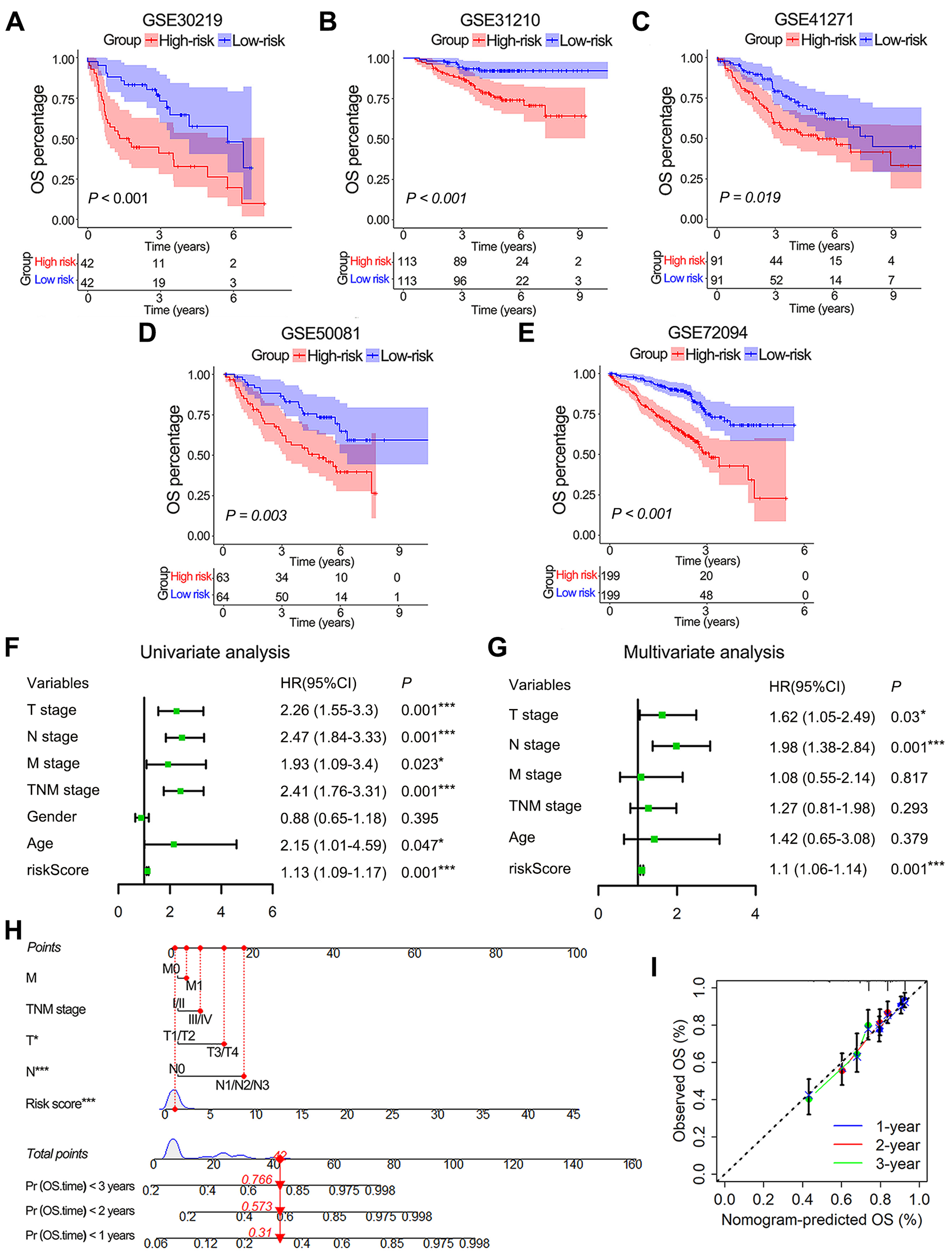

Supplement: Supplementary file 4 — Supplementary Material 4 [file 12935_2024_3382_MOESM4_ESM.tif]

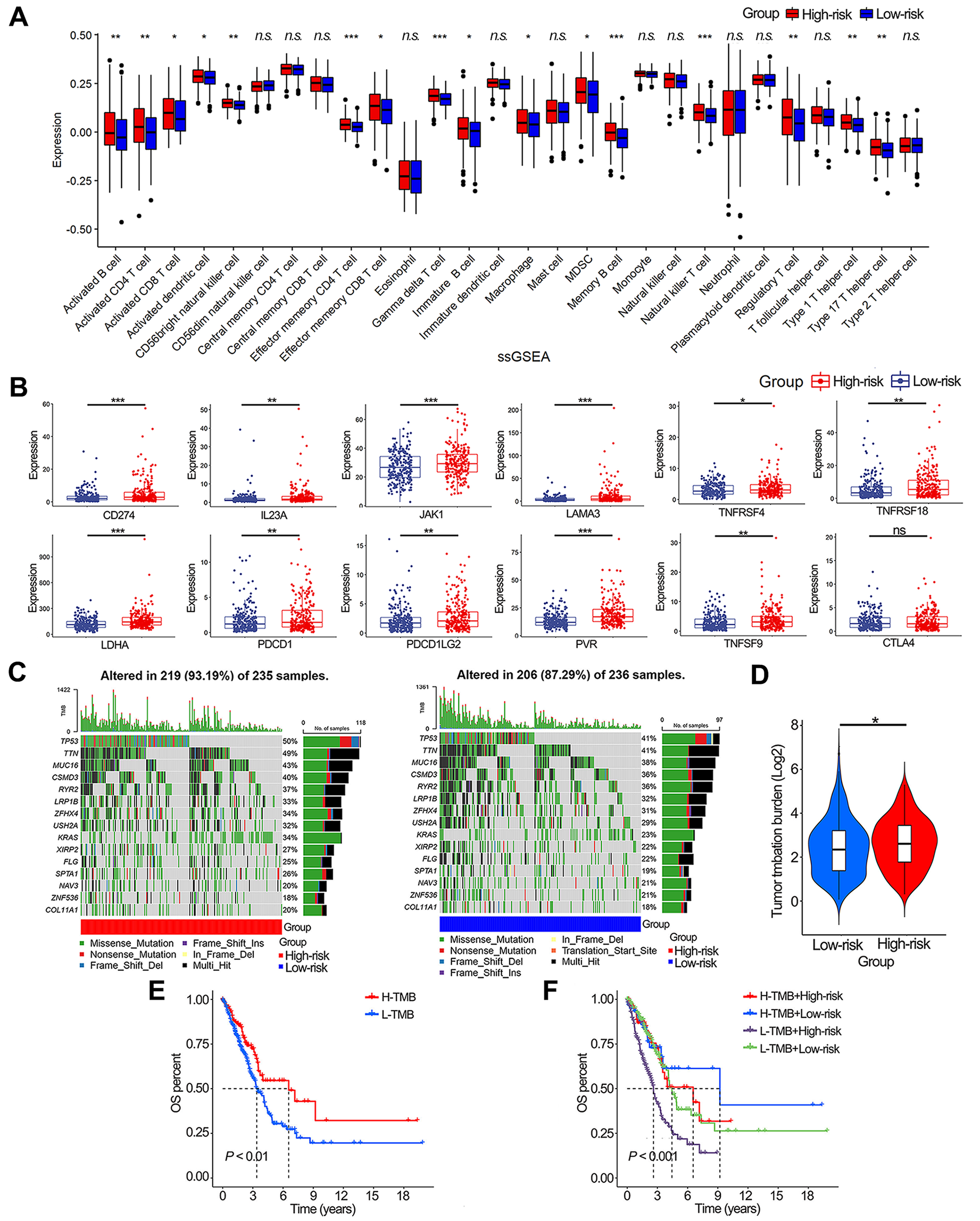

Supplement: Supplementary file 5 — Supplementary Material 5 [file 12935_2024_3382_MOESM5_ESM.tif]

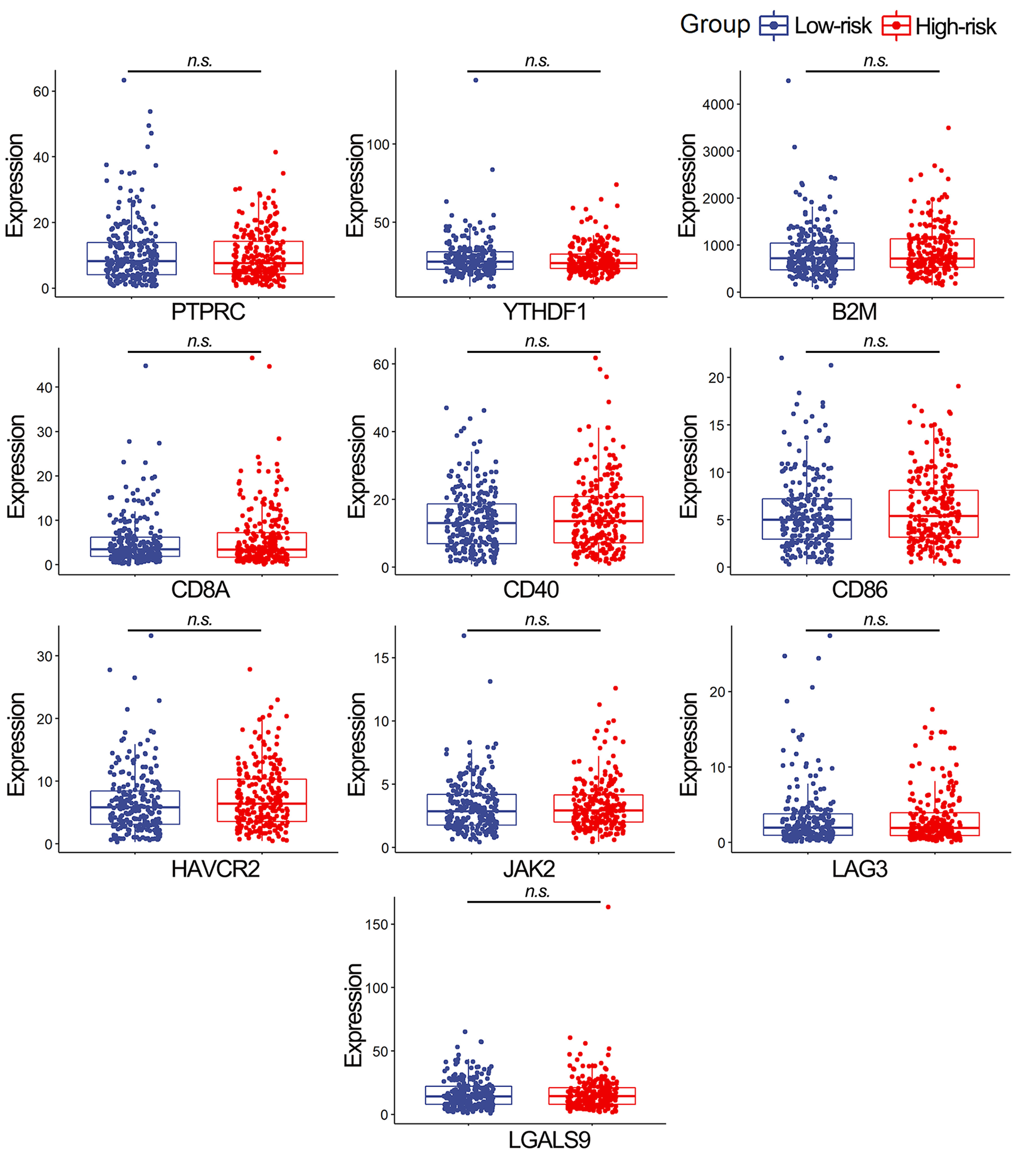

Supplement: Supplementary file 6 — Supplementary Material 6 [file 12935_2024_3382_MOESM6_ESM.tif]

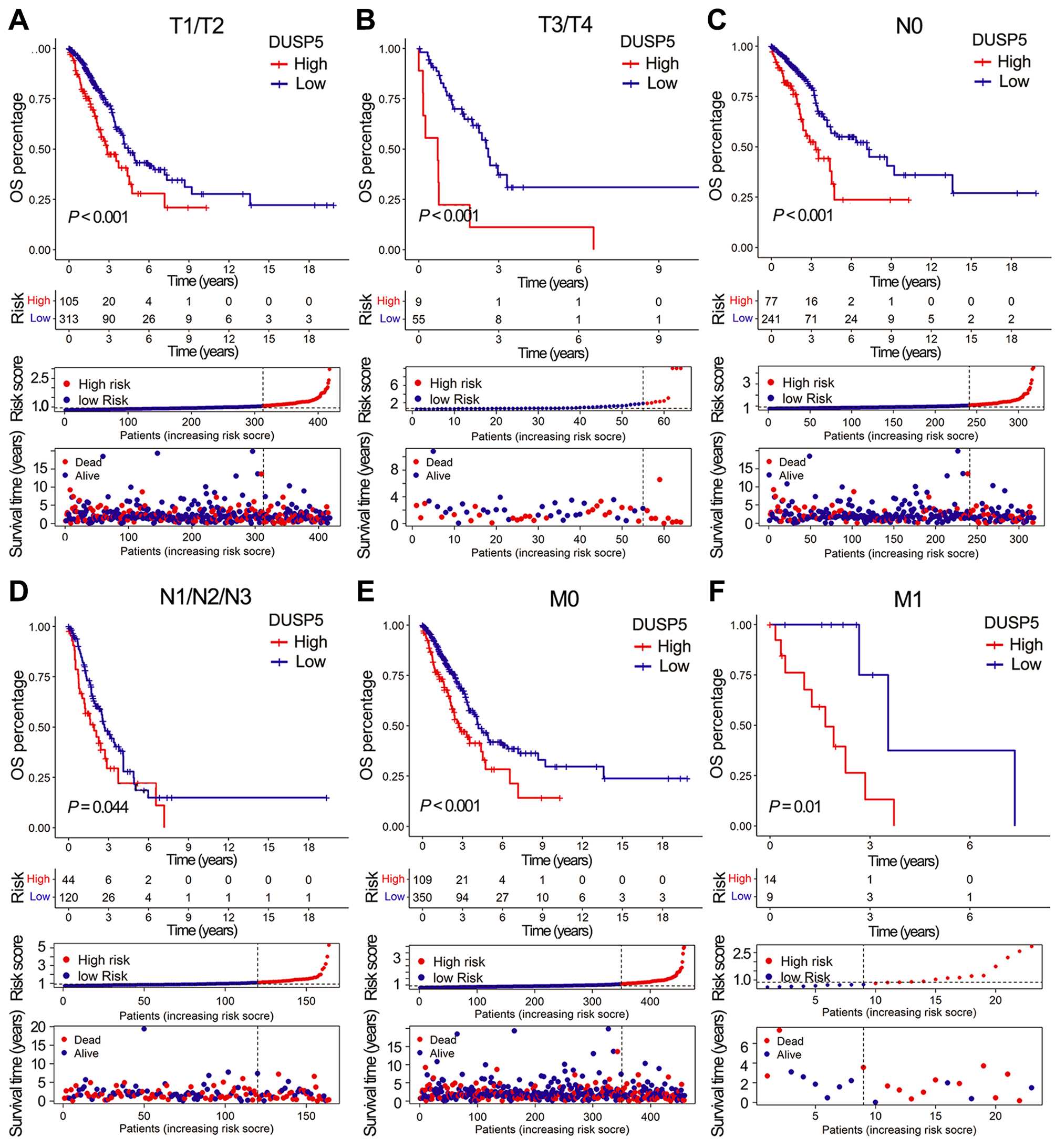

Supplement: Supplementary file 7 — Supplementary Material 7 [file 12935_2024_3382_MOESM7_ESM.tif]

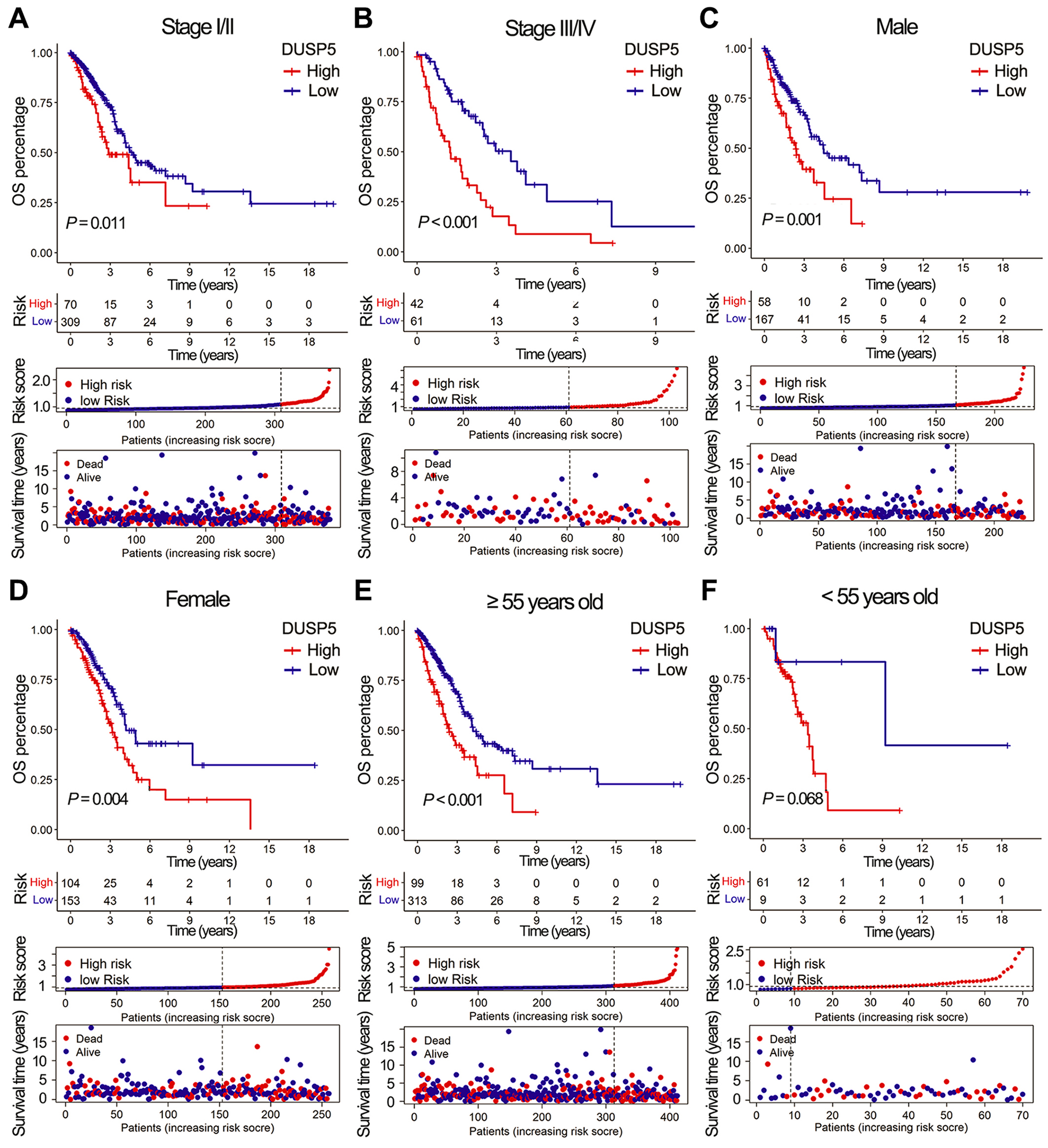

Supplement: Supplementary file 8 — Supplementary Material 8 [file 12935_2024_3382_MOESM8_ESM.tif]

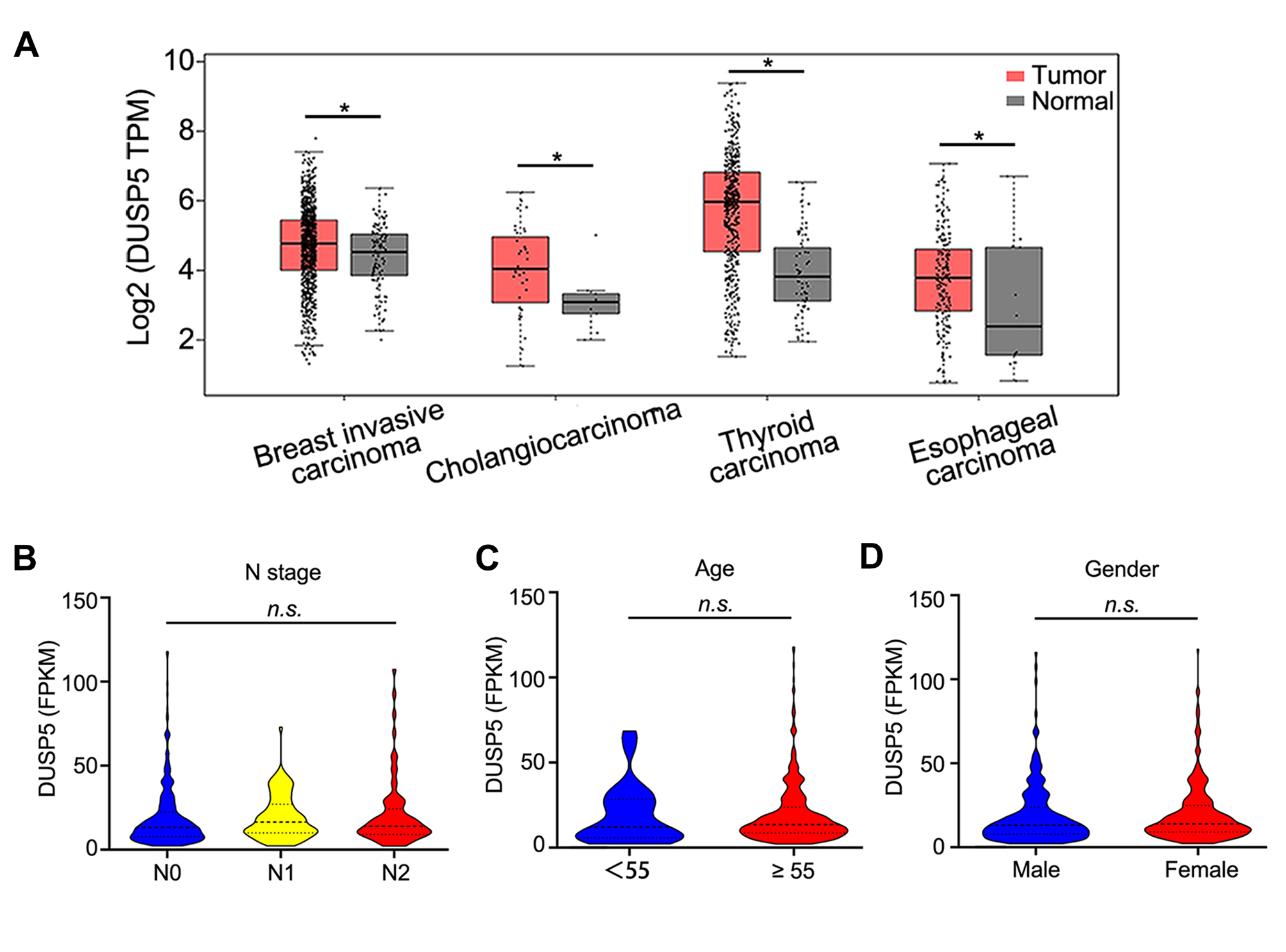

Supplement: Supplementary file 9 — Supplementary Material 9 [file 12935_2024_3382_MOESM9_ESM.tif]

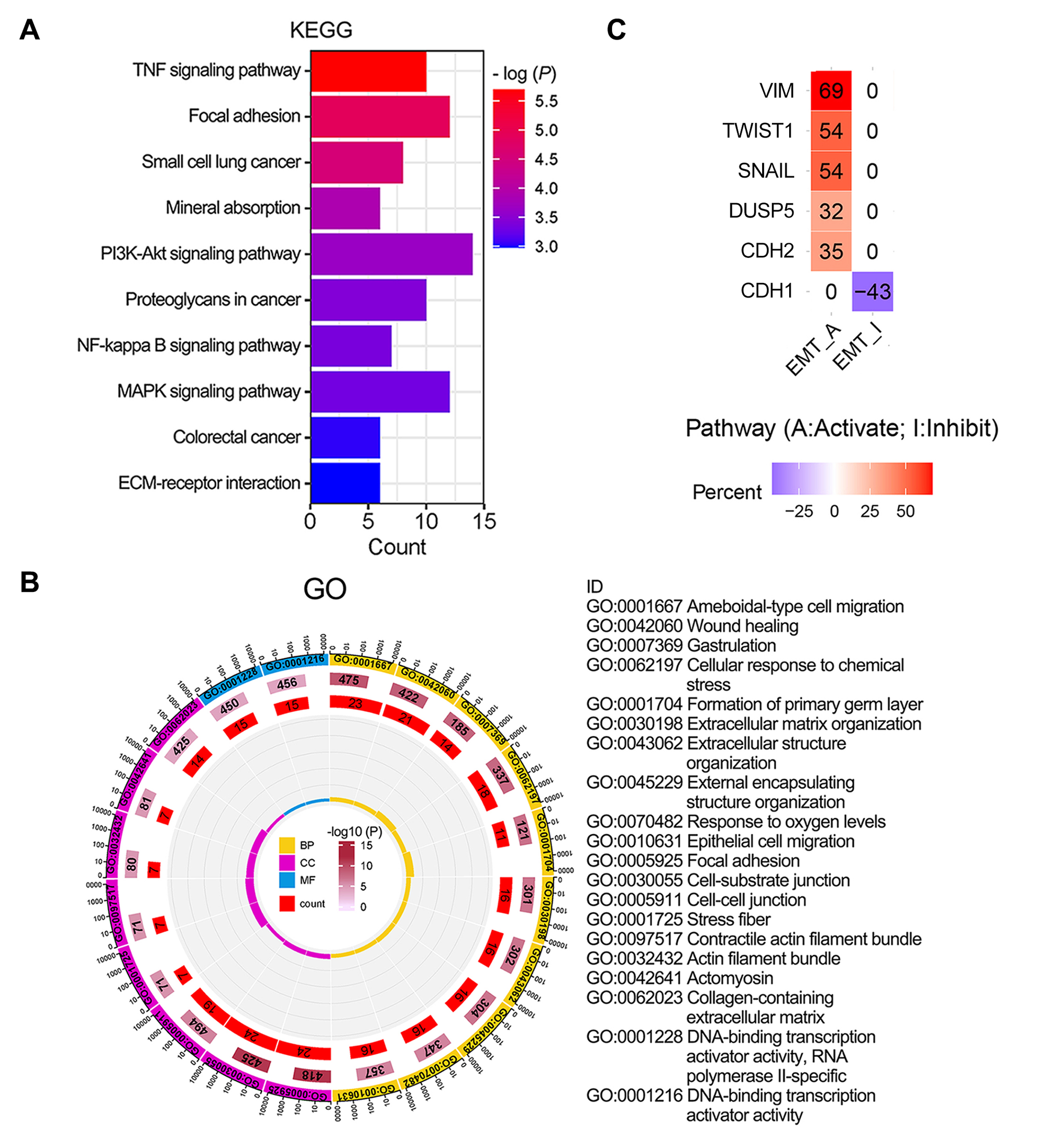

Supplement: Supplementary file 10 — Supplementary Material 10 [file 12935_2024_3382_MOESM10_ESM.tif]

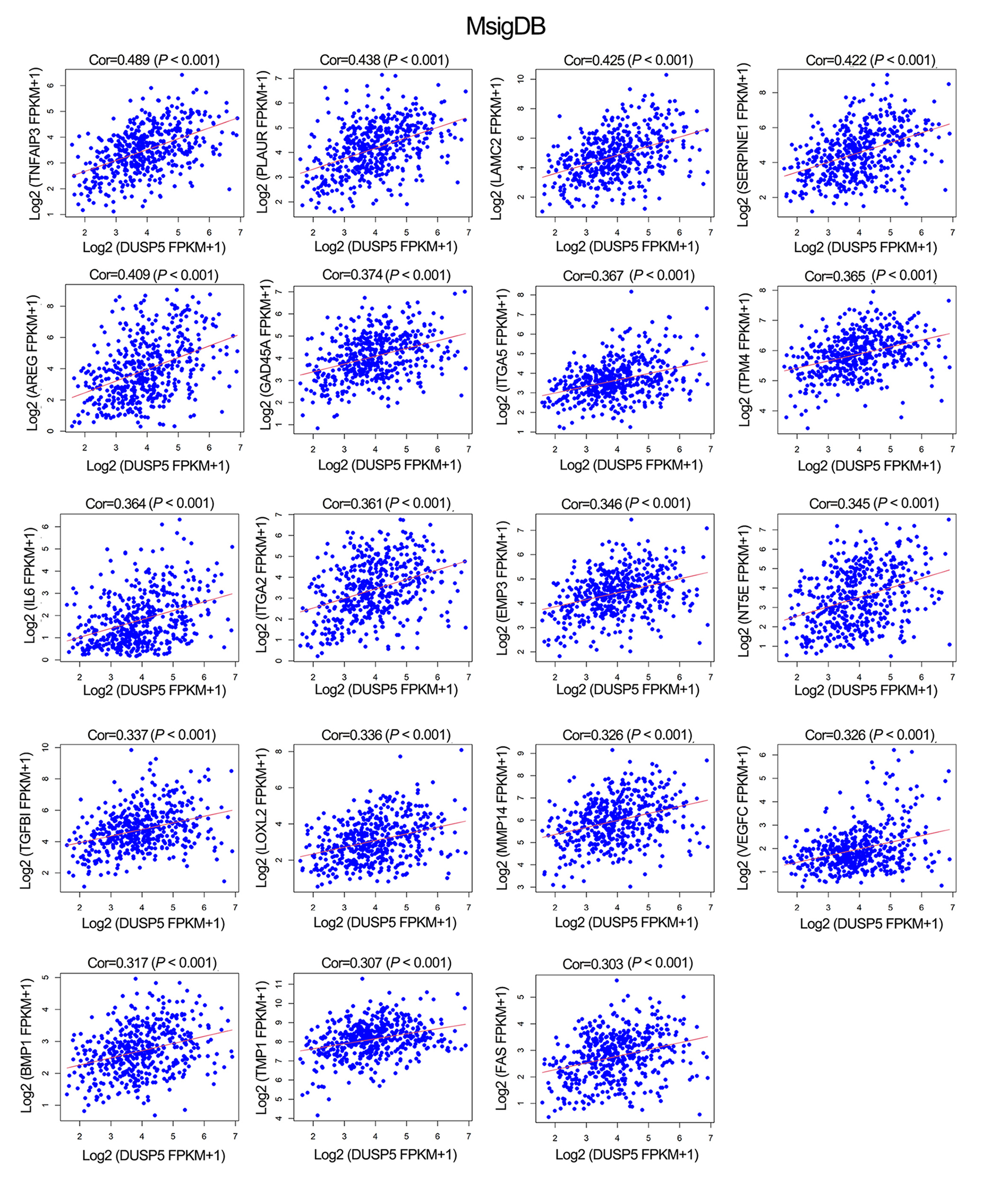

Supplement: Supplementary file 11 — Supplementary Material 11 [file 12935_2024_3382_MOESM11_ESM.tif]

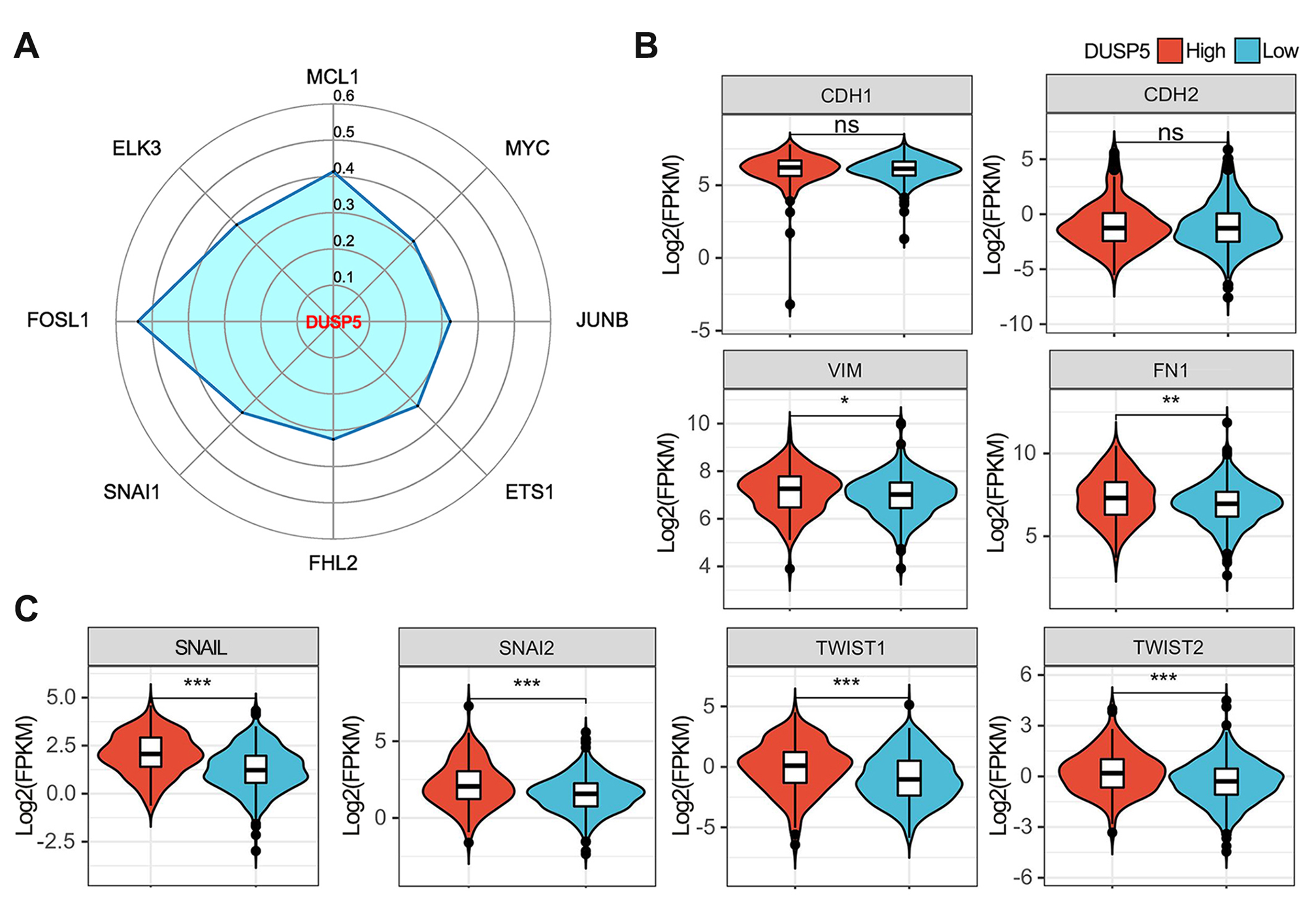

Supplement: Supplementary file 12 — Supplementary Material 12 [file 12935_2024_3382_MOESM12_ESM.tif]

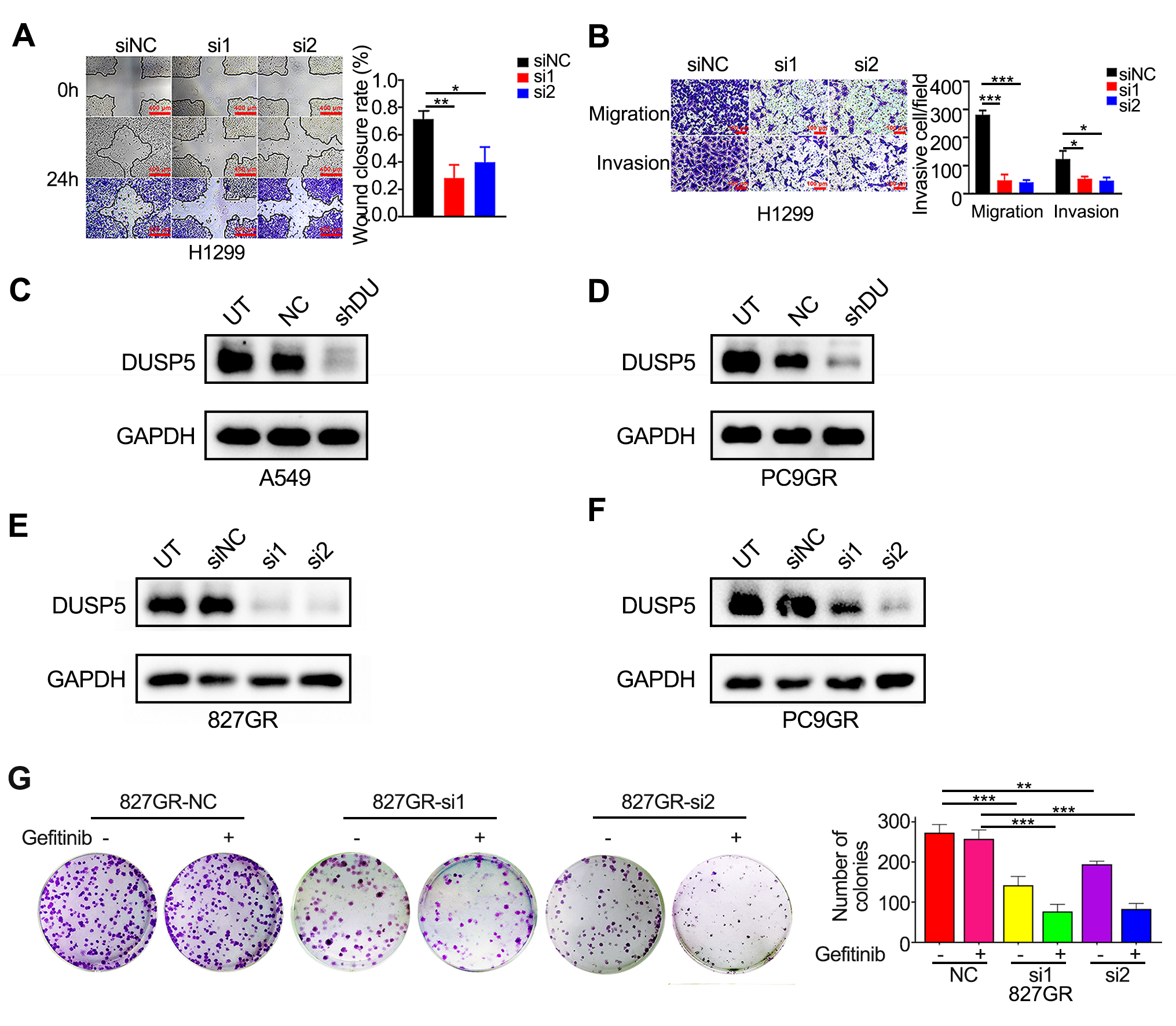

Supplement: Supplementary file 13 — Supplementary Material 13 [file 12935_2024_3382_MOESM13_ESM.tif]

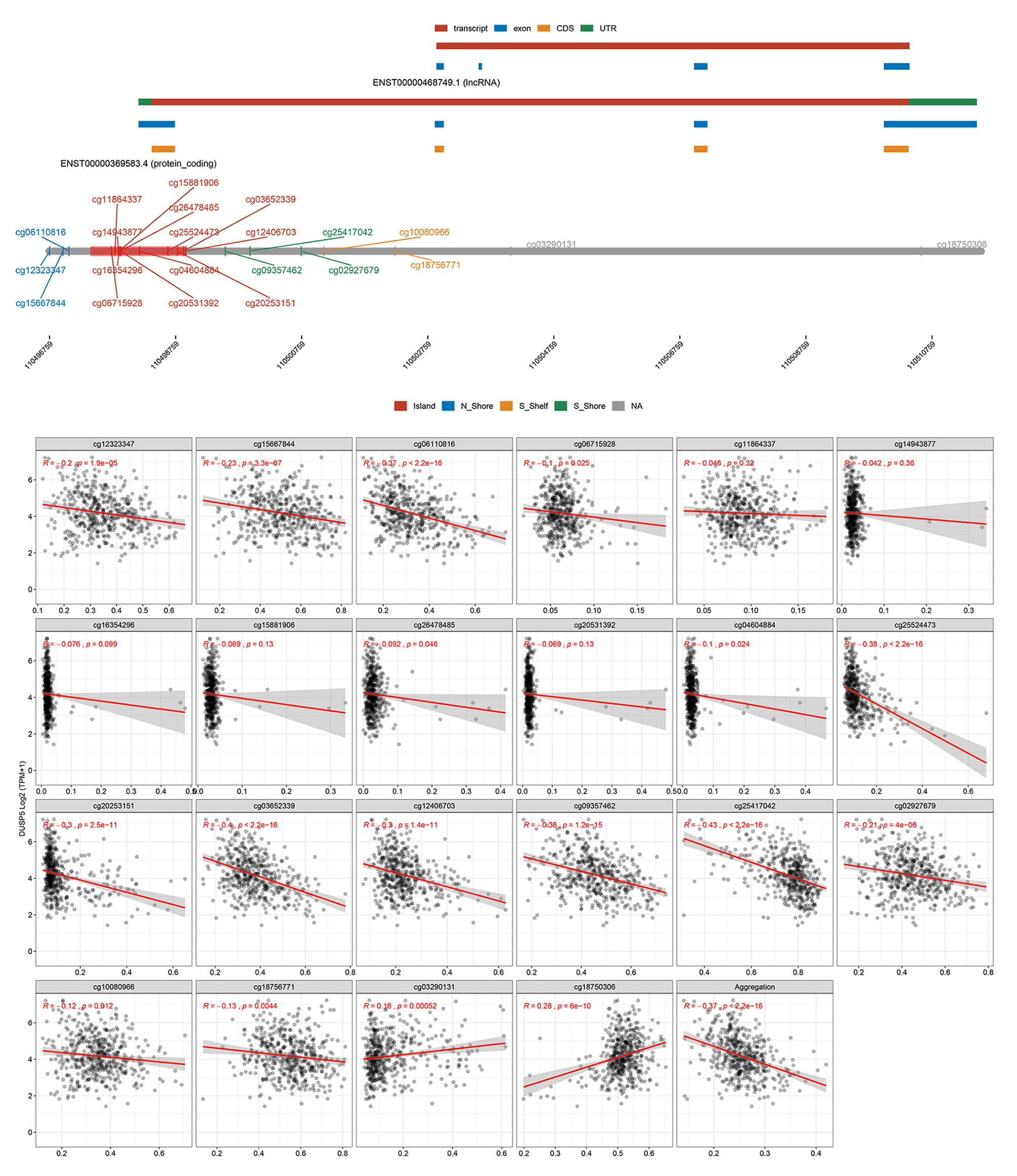

Supplement: Supplementary file 14 — Supplementary Material 14 [file 12935_2024_3382_MOESM14_ESM.tif]
