# Supplementary material for: DUSP5 regulated by YTHDF1-mediated m6A modification promotes epithelial-mesenchymal transition and EGFR-TKI resistance via the TGF-β/Smad signaling pathway in lung adenocarcinoma
Source: Cancer Cell Int. 2024 Jun 13;24:208. doi: 10.1186/s12935-024-03382-6 (PMC11177384; doi:10.1186/s12935-024-03382-6)
Supplement: Supplementary file 17 — Supplementary Material 17 [file 12935_2024_3382_MOESM17_ESM.docx]

**
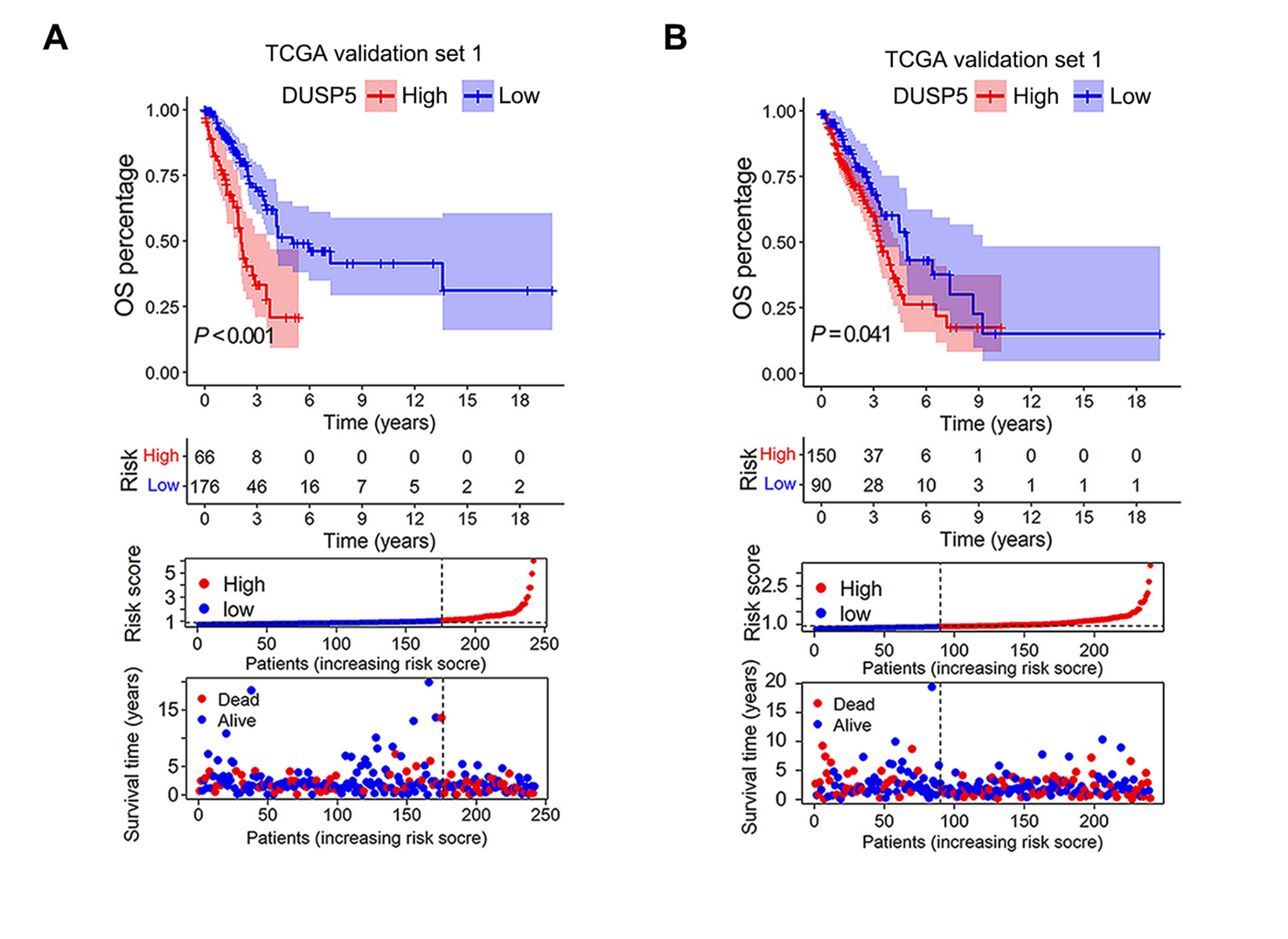
**

**Figure S1.** **Prognostic significance of DUSP5 in two validation sets from TCGA dataset.** (A-B) OS plot of DUSP5, distribution of patients and survival status in TCGA validation set 1 (A) and TCGA validation set 2 (B).

**
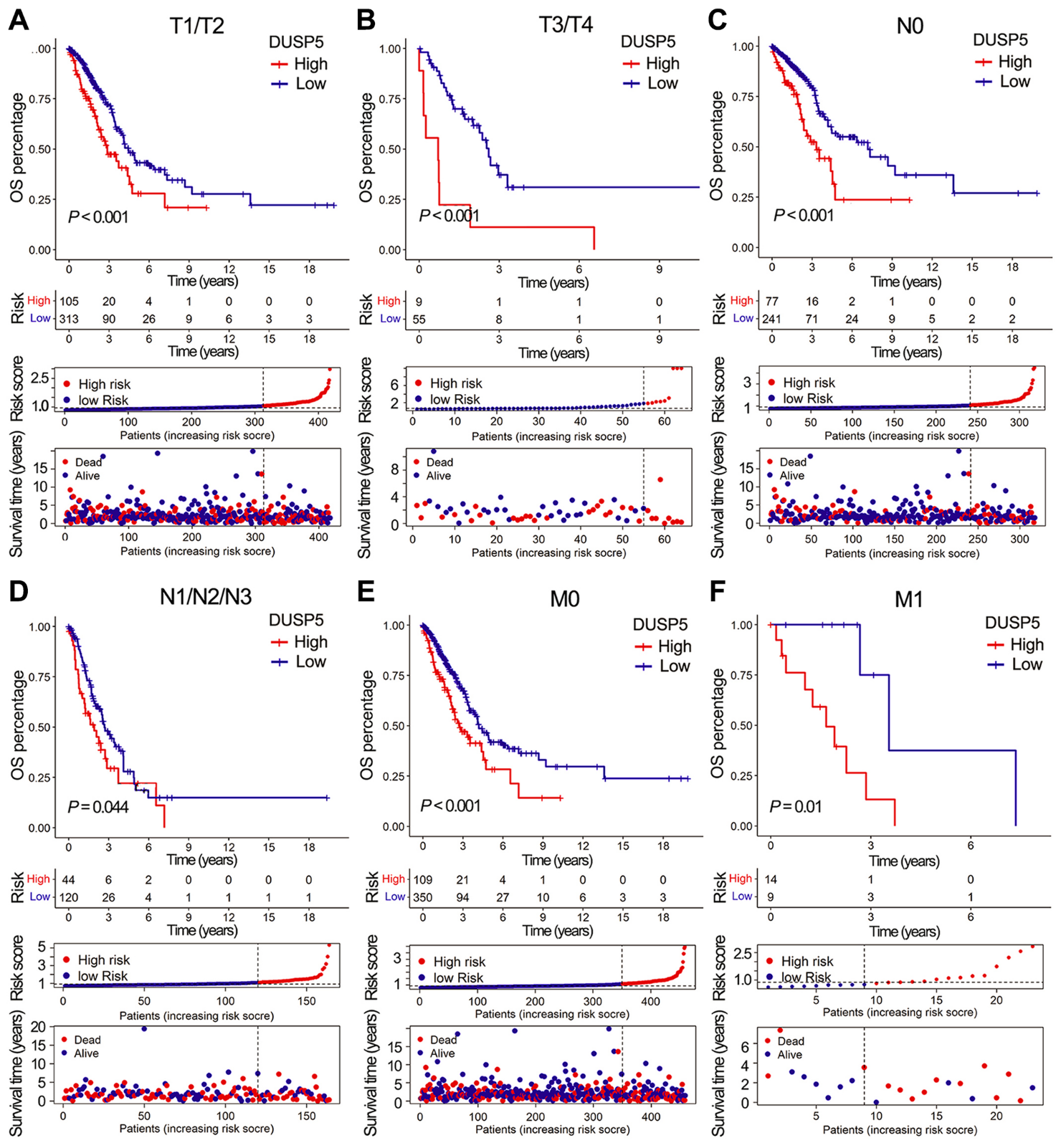
**

**Figure S2.** **Prognostic value of DUSP5 using a clinical parameters-stratified analysis.** (A-F) Kaplan–Meier overall survival plot of DUSP5 by using a clinical parameters-stratified analysis including T, N, and M stage.

**
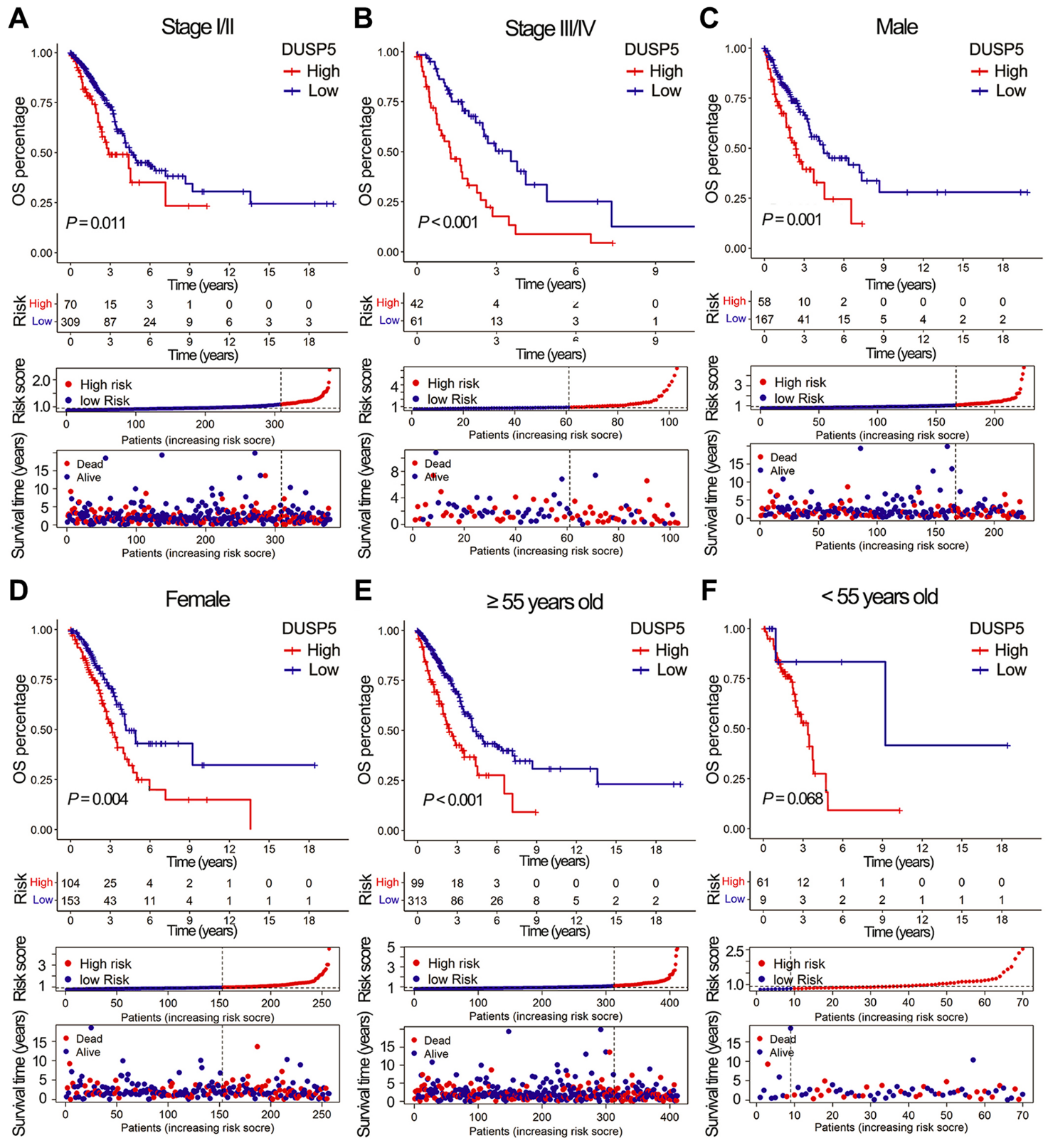
**

**Figure S3.** **Prognostic value of DUSP5 using a clinical parameters-stratified analysis. (A-F)** Kaplan–Meier overall survival plot of DUSP5 by using a clinical parameters-stratified analysis including TNM stage, gender and age.

**
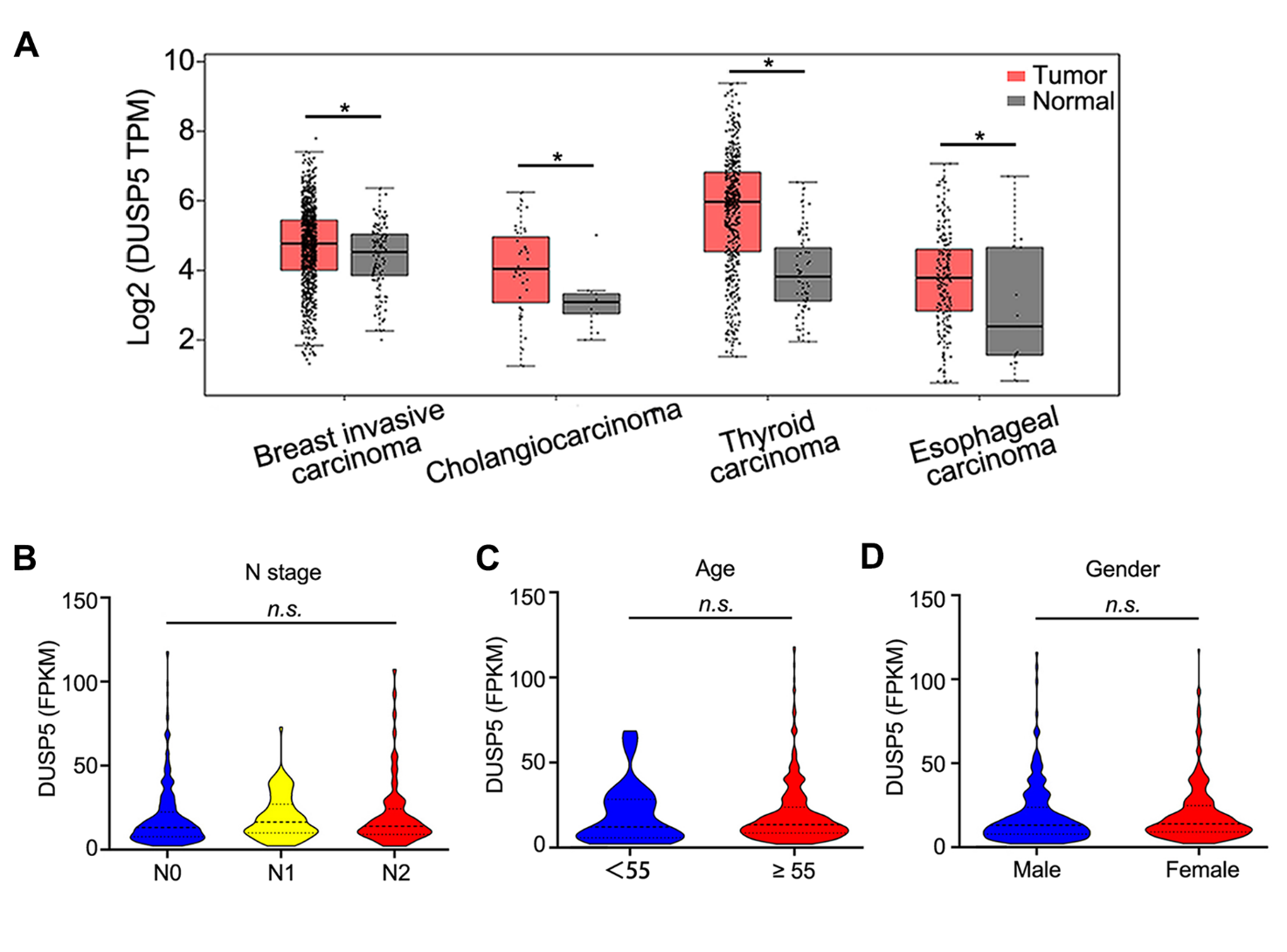
**

**Figure S4.** **The levels of DUSP5 expression in other cancers.** (A) Expression of DUSP5 across invasive breast carcinoma, cholangiocarcinoma, thyroid carcinoma and esophageal carcinoma. Red box: tumor samples, gray box: normal samples. (B-D) The levels of DUSP5 expression were shown according to different N stage, age and gender.

**
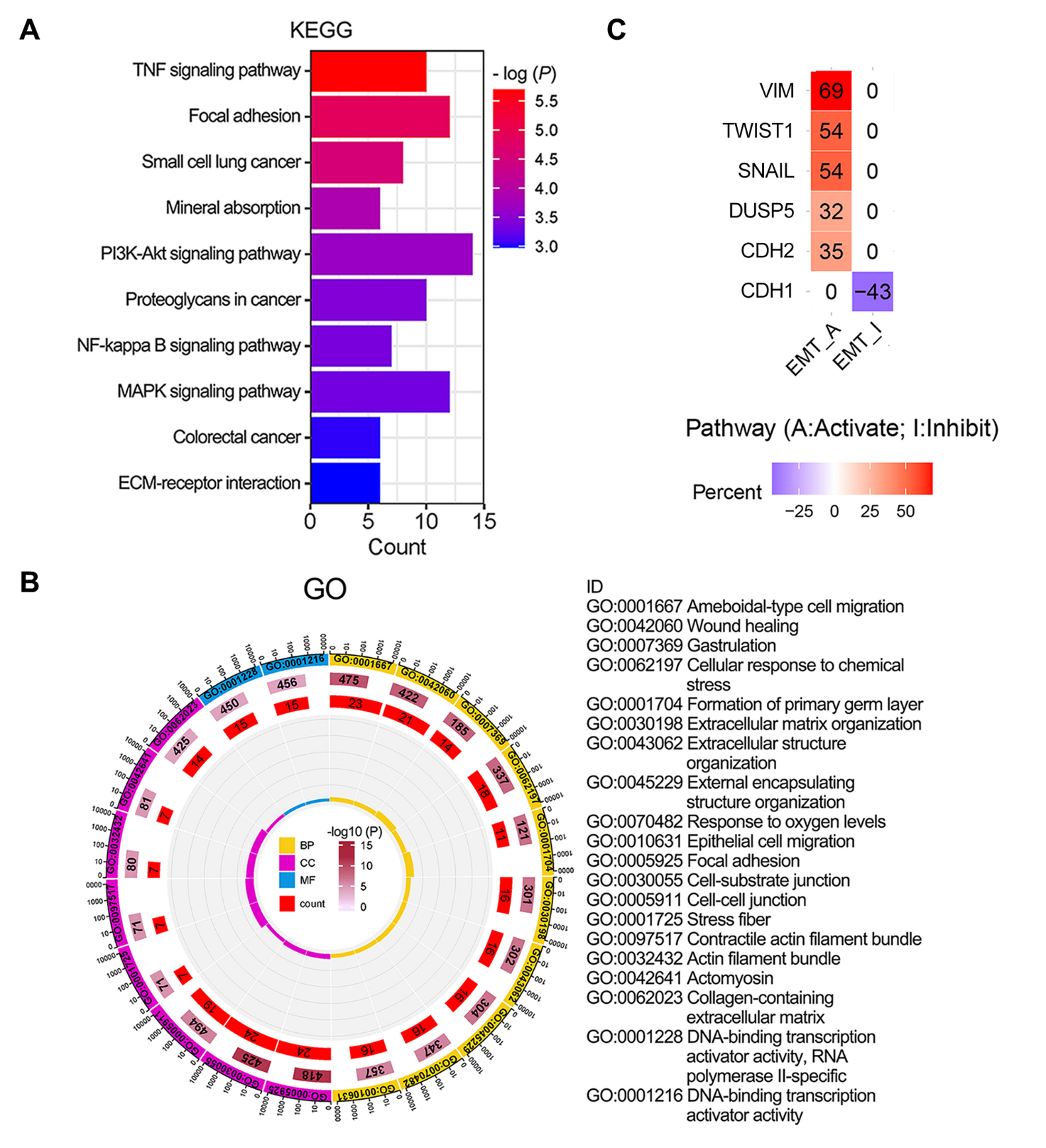
**

**Figure S5.** **Functional enrichment analysis and EMT pathway analysis** **in GSCALite.** (A) KEGG enrichment analysis results were shown by R language. (B) GO enrichment analysis results were shown in the top 10 biological processes including 8 molecular functions and 2 cellular components. (C) The squares represent the associations between the gene, including CDH1, CDH2, DUSP5, SNAIL, TWIST1 and VIM, and the EMT pathway.

**
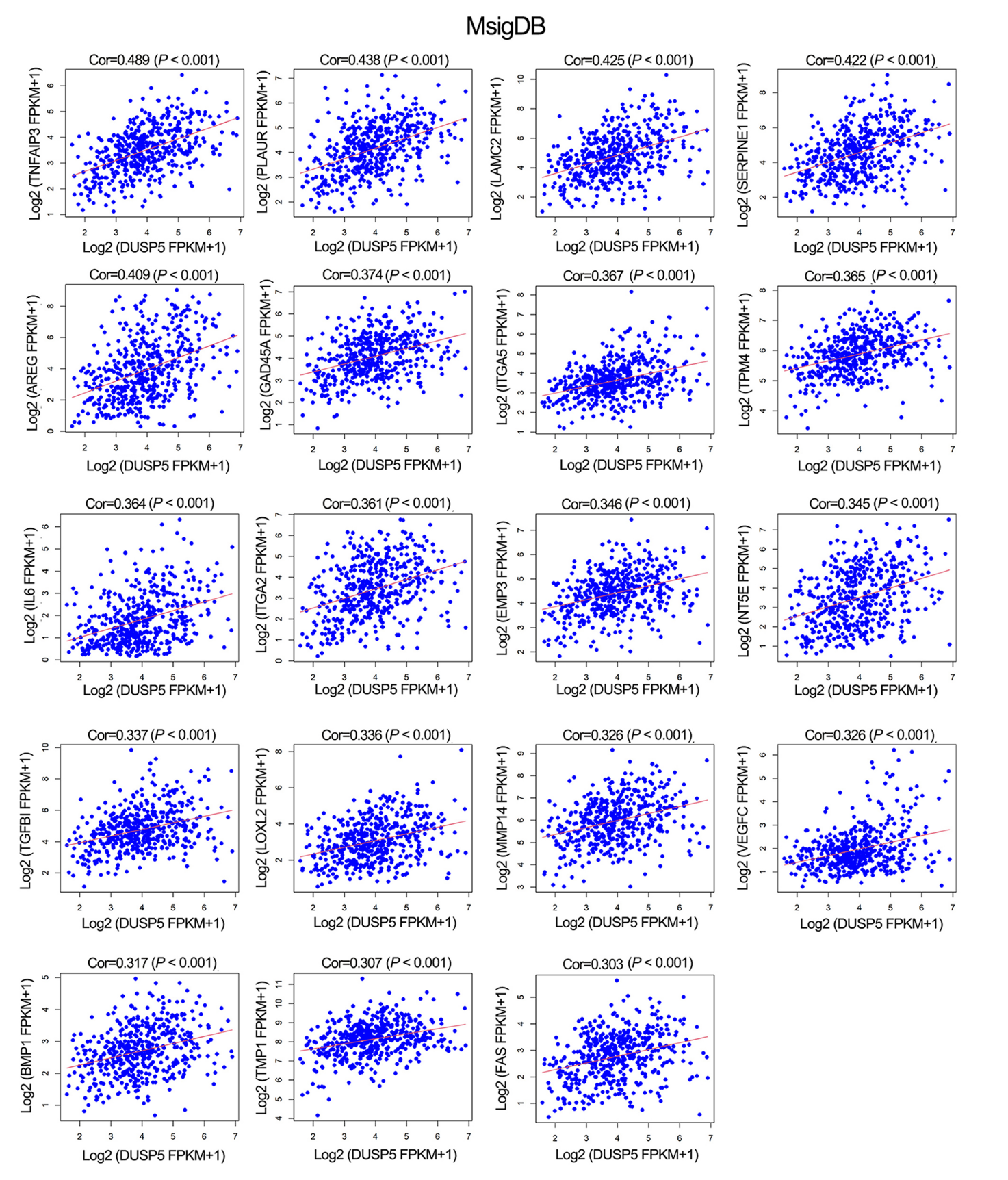
**

**Figure S6.** **The correlations between DUSP5 and EMT-related genes.** The plots were used to exhibit the correlation of DUSP5 with EMT-related genes (at least *P* < 0.05, Cor > 0.3) from MsigDB. Cor: correlation coefficient.

**
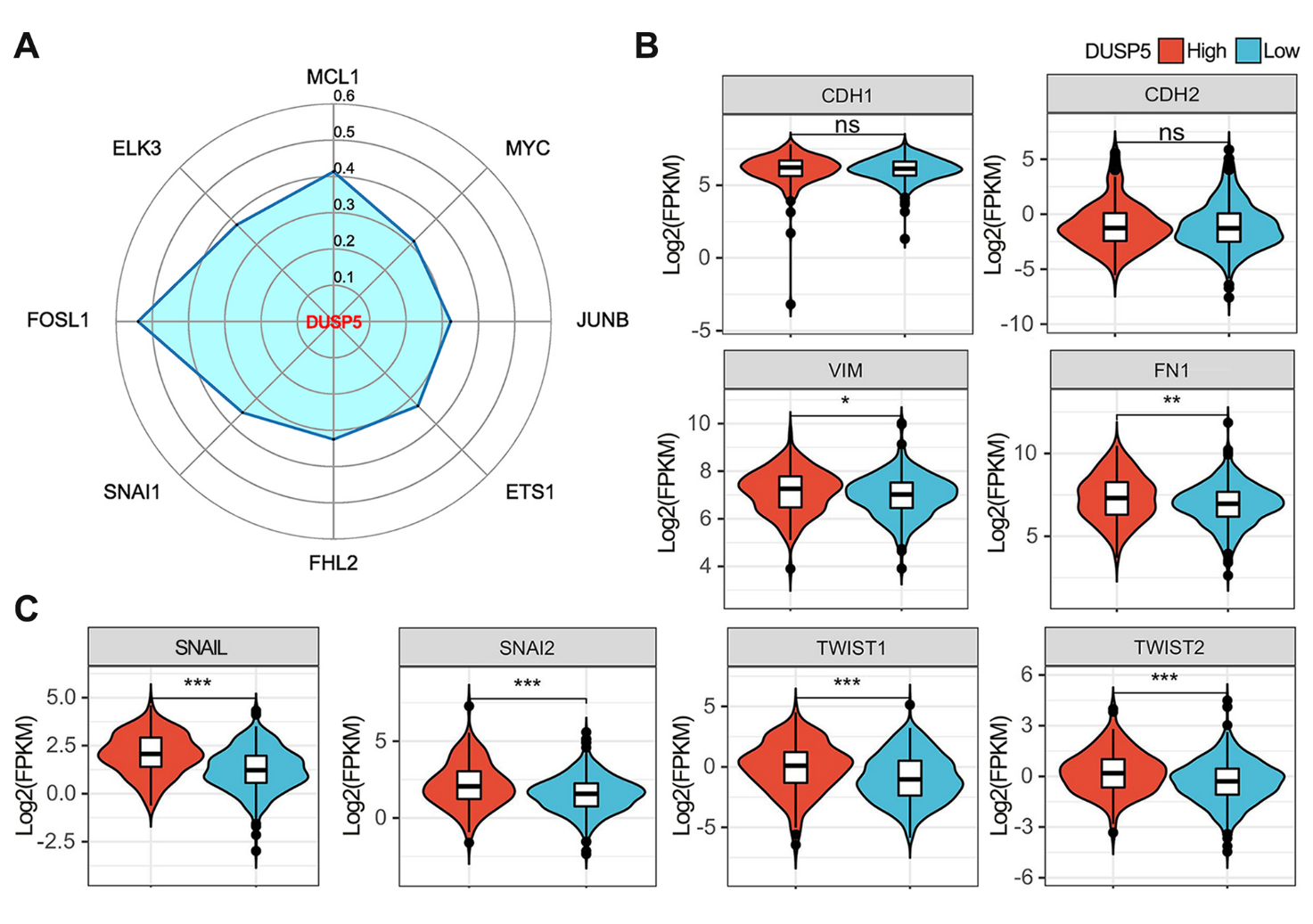
**

**Figure S7.** **The relationships between DUSP5 and EMT-related markers from dbEMT.** (A) The correlation of oncogenic EMT-related genes from dbEMT with DUSP5 in radar charts. (B-C) Expression of EMT-associated markers between two groups according to DUSP5 expression. **P* < 0.05, ***P* < 0.01, ****P* < 0.001.

**
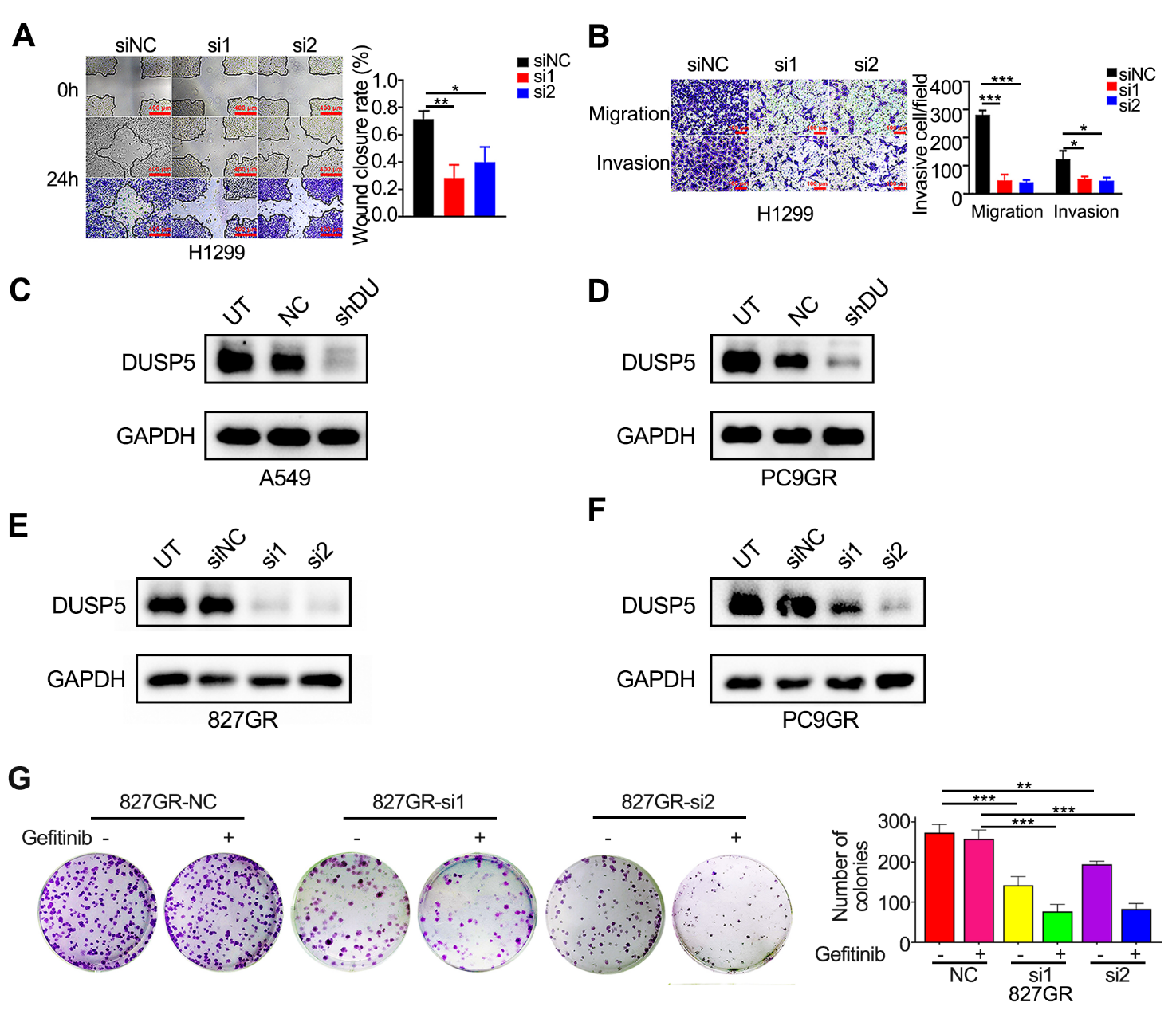
**

**Figure S8.** **DUSP5 knockdown cell lines were validated.** (A-B) Cell migratory and invasive capability was elucidated by Wound-healing and Transwell assays in H1299-derived cell lines. (C-D) Expression of DUSP5 in A549 (C) and PC9GR (D) cells transfected with shNC (NC) or shDUSP5 (shDU). (E-F) The levels of DUSP5 expression in LUAD cells after transfected with siNC and siDUSP5 (si1 and si2) were examined by Western blotting. (G) The proliferation of gefitinib-treated or untreated 827GR-derived cells was detected by colony formation assay.

**
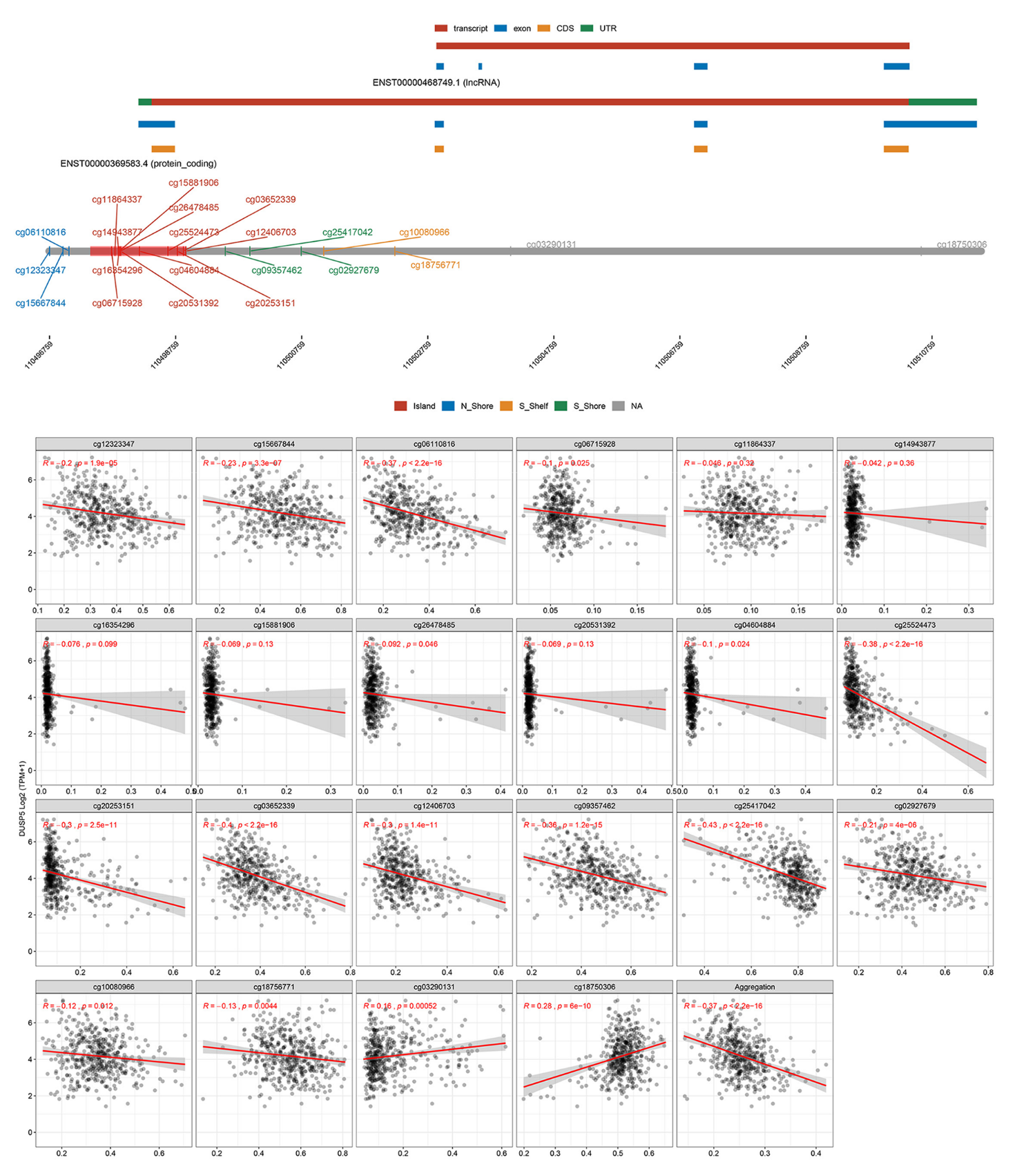
**

**Figure S9. Schematic diagram of DUSP5 methylation sites and the correlations between various CpG sites and DUSP5 expression.**

**
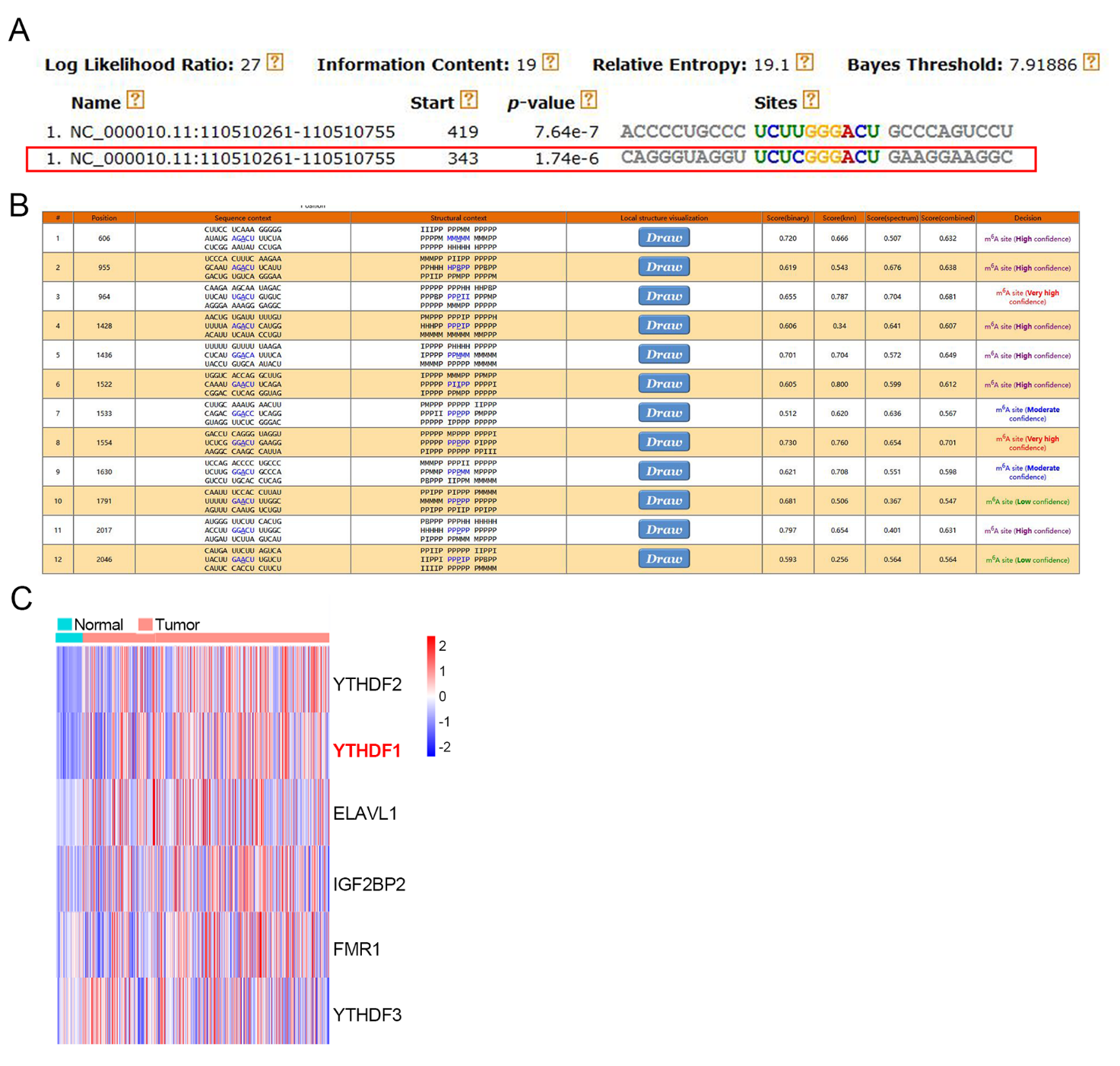
**

**Figure S10. Bioinformatic prediction of m6A modification of DUSP5 mRNA and screening of YTHDF1** (A-C)**.**

**
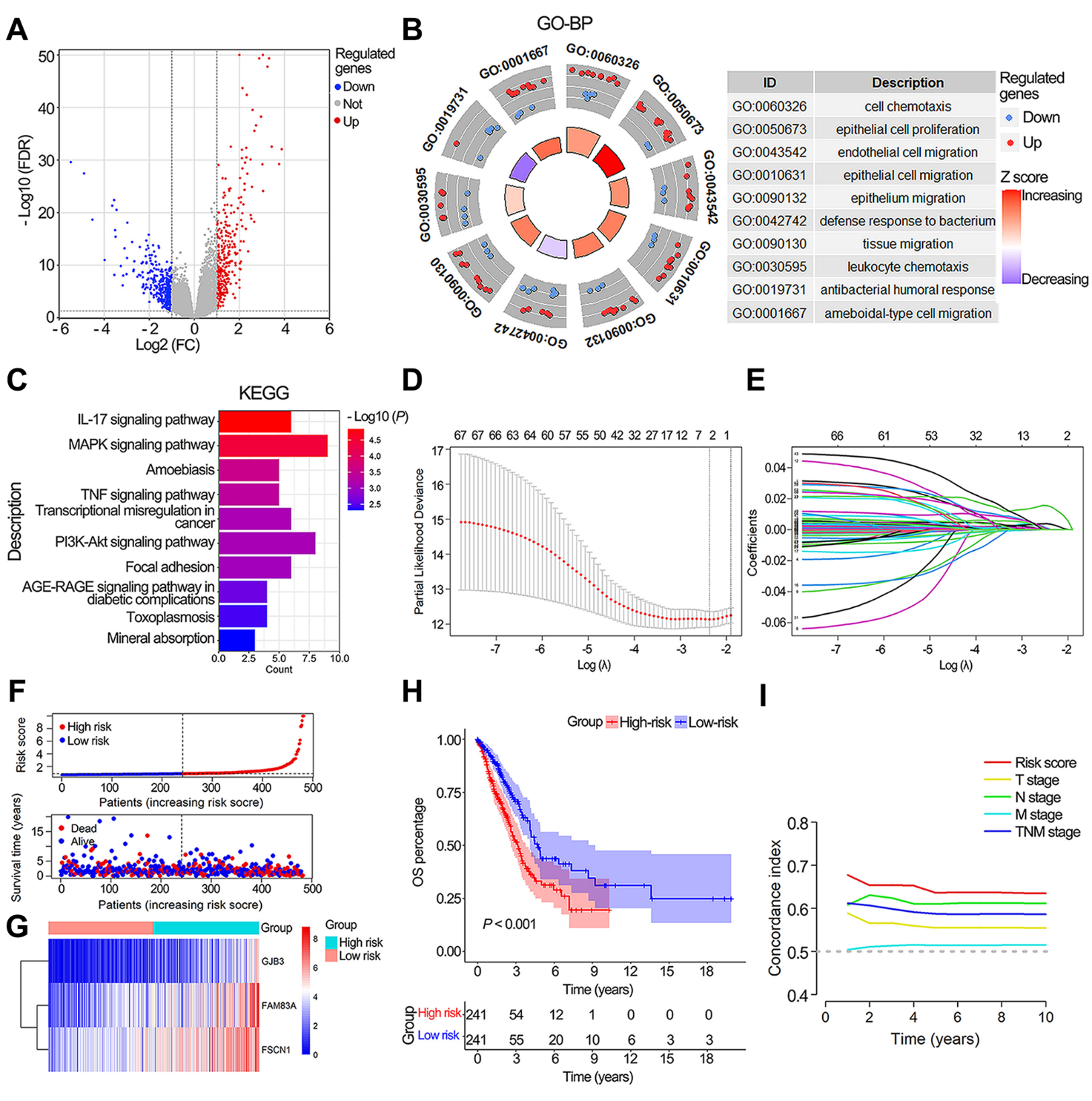
**

**Figure S11. The DUSP5-originated genomic model was built.** (A) Volcano plots exhibited DEGs between TCGA samples expressing high and low levels of DUSP5. (B) The circle heat map showed items of GO functional enrichment in the top 10. (C) KEGG enrichment analysis was shown in the top 10. (D, E) Regression was carried out using the minimum criteria, three genes with predictive value profiles using LASSO (GJB3, FAM83A and FSCN1). (F) Distribution of risk scores and survival status of three genes. (G) The heatmap of three potential genes’ expression. (H) OS of patients according to the two groups. (I) Concordance index of different prognostic factors and model’s risk score. FC: fold change.

**
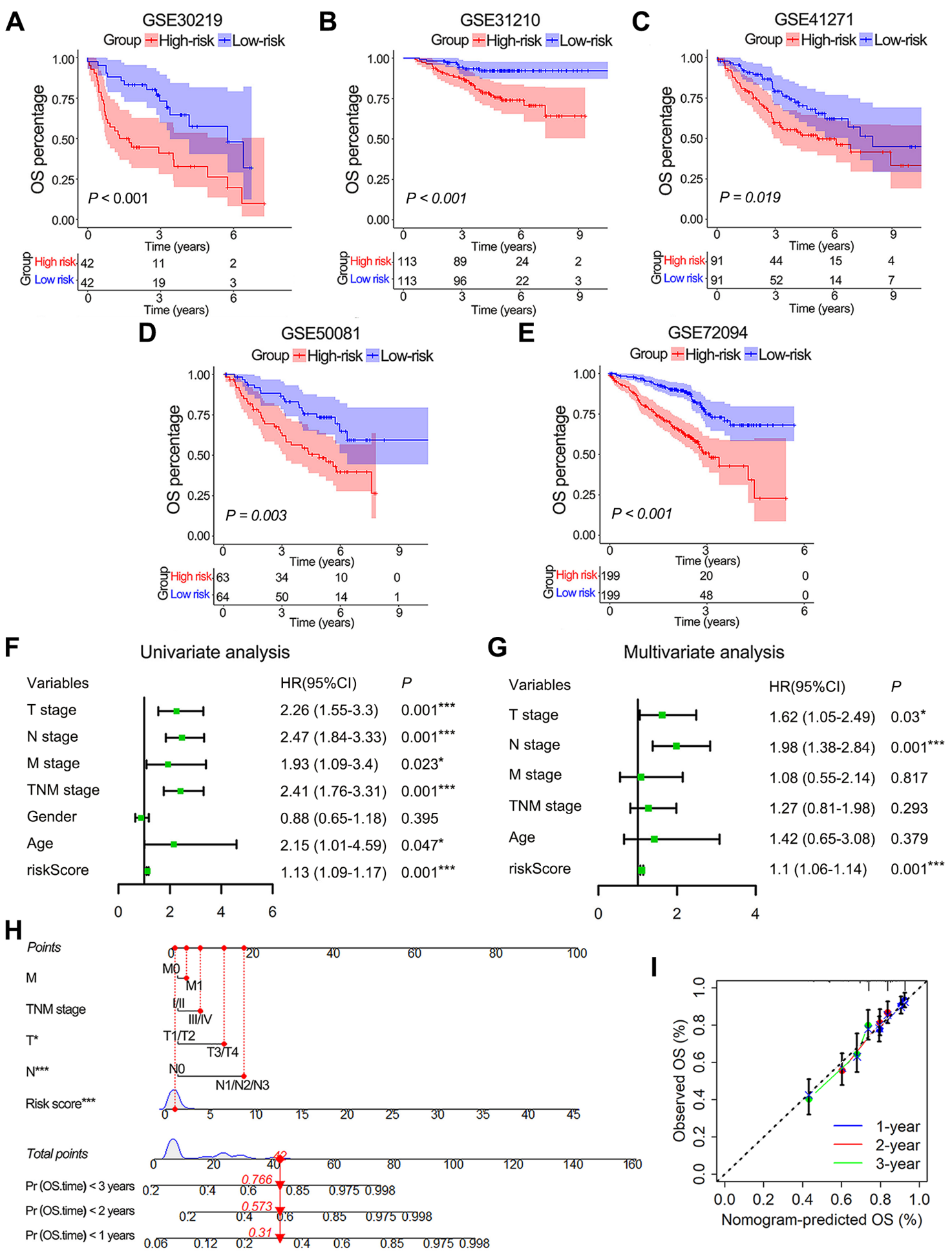
**

**Figure S12.** **The** **validation of the DUSP5-originated genomic model’s ability to predict poor outcomes.** (A-E) OS of high-risk and low-risk groups in GSE30219, GSE31210, GSE41271, GSE50081 and GSE72094 datasets. (F-G) Univariate and multivariate Cox regression analysis of genomic model and clinical characteristics using a forest map. (H) The nomogram predicted the probability of OS. (I) The calibration plot accessed the actual survival and predicted survival probability of OS. **P* < 0.05, ***P* < 0.01, ****P* < 0.001.

**
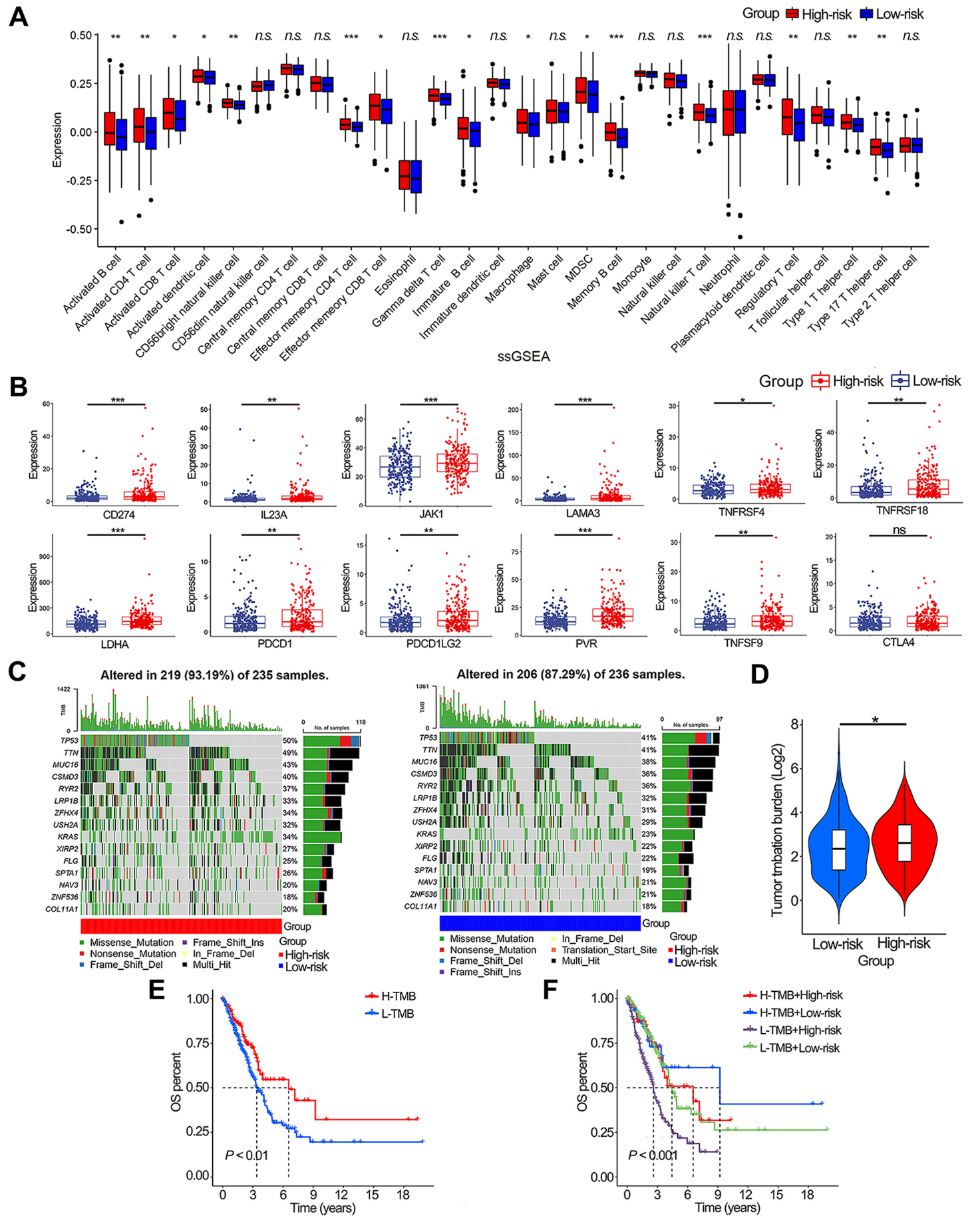
**

**Figure S13. Immune features and TMB in two groups revealed by DUSP5-originated genomic model.** (A) Analysis of infiltration levels of immune cells in two groups using ssGSEA. (B) Immune checkpoint genes differed between two groups. (C) The features of somatic mutations in the two groups are depicted in waterfall plots. (D) The variation of TMB between high-risk and low-risk groups. (E) Survival rates in groups with high and low TMB. (F) OS of four patient groups based on TMB status and DUSP5-originated genomic model’s risk score.

**
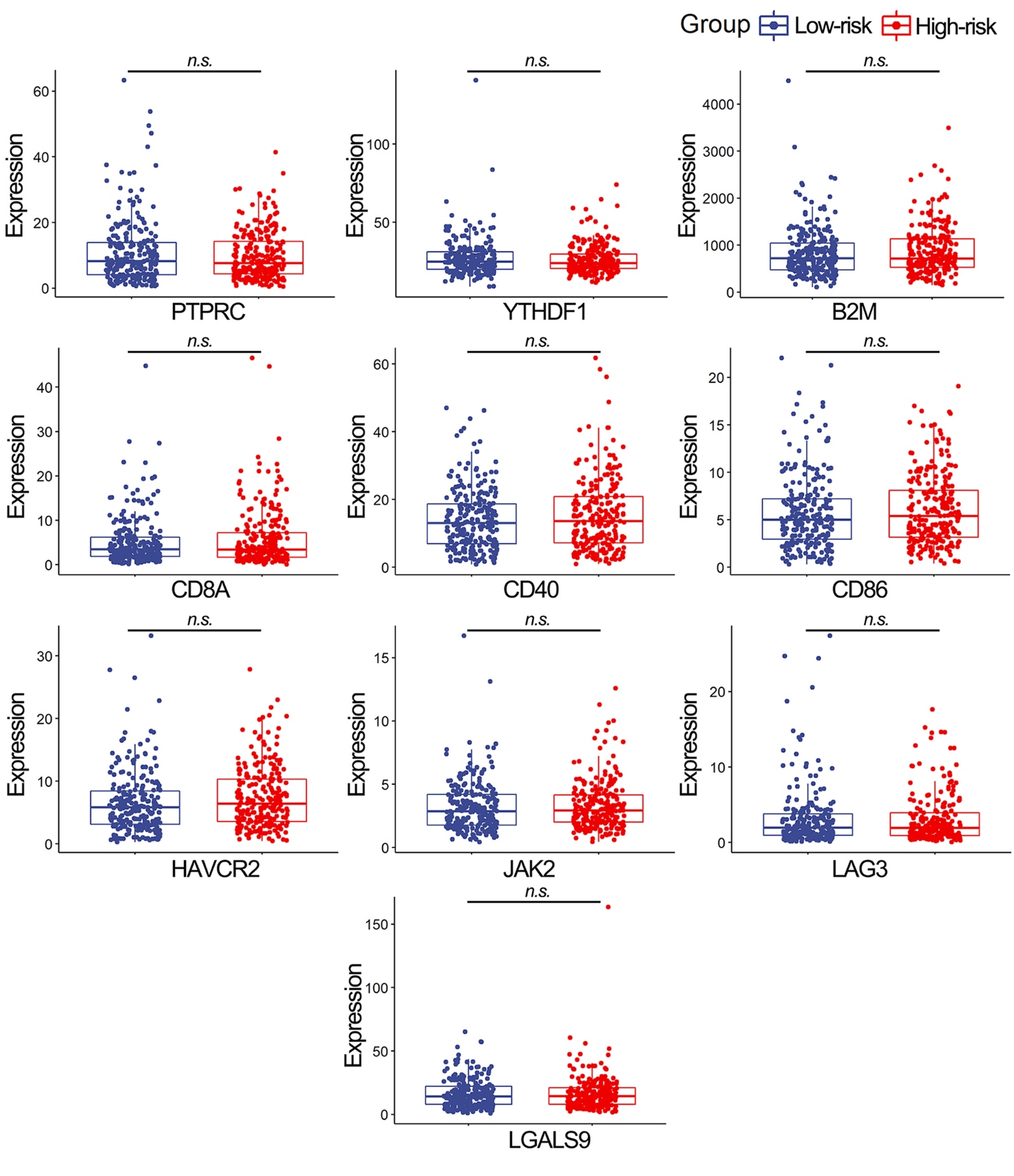
**

**Figure S14. The expression of the immune checkpoint genes in two groups was revealed by the DUSP5-originated genomic model.**

**Table S1. The information of two validation sets from the TCGA database.**

**Table S2. Univariate-Cox regression of DUSP5-originated DEGs in TCGA.**
